# Comparative and Evolutionary Analysis of the HES/HEY Gene Family Reveal Exon/Intron Loss and Teleost Specific Duplication Events

**DOI:** 10.1371/journal.pone.0040649

**Published:** 2012-07-13

**Authors:** Mi Zhou, Jun Yan, Zhaowu Ma, Yang Zhou, Nibras Najm Abbood, Jianfeng Liu, Li Su, Haibo Jia, An-Yuan Guo

**Affiliations:** 1 Hubei Bioinformatics and Molecular Imaging Key Laboratory, Department of Biomedical Engineering, College of Life Science and Technology, Huazhong University of Science and Technology, Wuhan, Hubei, People’s Republic of China; 2 Key Laboratory of Molecular Biophysics of the Ministry of Education, Sino-France Laboratory for Drug Screening, College of Life Science and Technology, Huazhong University of Science and Technology, Wuhan, Hubei, People’s Republic of China; 3 Key Laboratory of Molecular Biophysics of Ministry of Education, College of Life Science and Technology, Center for Human Genome Research, Huazhong University of Science and Technology, Wuhan, Hubei, People’s Republic of China; University of Chicago, United States of America

## Abstract

**Background:**

HES/HEY genes encode a family of basic helix-loop-helix (bHLH) transcription factors with both bHLH and Orange domain. HES/HEY proteins are direct targets of the Notch signaling pathway and play an essential role in developmental decisions, such as the developments of nervous system, somitogenesis, blood vessel and heart. Despite their important functions, the origin and evolution of this HES/HEY gene family has yet to be elucidated.

**Methods and Findings:**

In this study, we identified genes of the HES/HEY family in representative species and performed evolutionary analysis to elucidate their origin and evolutionary process. Our results showed that the HES/HEY genes only existed in metazoans and may originate from the common ancestor of metazoans. We identified HES/HEY genes in more than 10 species representing the main lineages. Combining the bHLH and Orange domain sequences, we constructed the phylogenetic trees by different methods (Bayesian, ML, NJ and ME) and classified the HES/HEY gene family into four groups. Our results indicated that this gene family had undergone three expansions, which were along with the origins of Eumetazoa, vertebrate, and teleost. Gene structure analysis revealed that the HES/HEY genes were involved in exon and/or intron loss in different species lineages. Genes of this family were duplicated in bony fishes and doubled than other vertebrates. Furthermore, we studied the teleost-specific duplications in zebrafish and investigated the expression pattern of duplicated genes in different tissues by RT-PCR. Finally, we proposed a model to show the evolution of this gene family with processes of expansion, exon/intron loss, and motif loss.

**Conclusions:**

Our study revealed the evolution of HES/HEY gene family, the expression and function divergence of duplicated genes, which also provide clues for the research of Notch function in development. This study shows a model of gene family analysis with gene structure evolution and duplication.

## Introduction

Notch signaling is a crucial cell signaling pathway in metazoan development, which has been shown to play an important role in regulating cellular identity, proliferation, differentiation and apoptosis [1]–[3]. Activation of Notch signaling pathway leads to nuclear translocation of Notch receptor intracellular domain (NIC), then NIC binds to CBF1 (RBPJ-k), and thereby activates transcription of effectors, for example, the HES and HEY genes [4], [5]. HES/HEY genes, as the direct targets and effectors of the Notch signaling pathway, encode a small family of basic helix-loop-helix (bHLH) transcription factors (TFs) that are related to the Drosophila hairy and Enhancer-of-split genes [4], [6]. In addition, there is some crosstalk between Notch and the BMP/TGF-beta, JAK-STAT, Ras, PI3K, ERK, HIF signaling pathways to enhance the activation of HES/HEY expression, suggesting that they can transduce and integrate signals from multiple important signaling pathways [7]–[11]. Furthermore, recent studies revealed that some members of HES/HEY act upstream or independent of, but not downstream of Notch during hematopoietic stem cell specification and hair cell differentiation [12], [13]. Considering the importance and divergence of HES/HEY genes in signaling pathways, exploring the evolution of this gene family will greatly help to elucidate their functions.

HES stand for those proteins which show homology to the Drosophila hairy and Enhancer of split basic helix-loop-helix (bHLH) proteins, the first reported proteins of these were the rat HES1/Hairy-like protein and several related proteins designated HES2-5 [14]. Since HES/HEY genes are bHLH TFs containing a bHLH DNA-binding domain. Besides that, they also contain a conserved Orange domain and possibly a conserved C-terminal tetrapeptide WRPW or YRPW motifs. They may form as either homodimers or heterodimers with other Hairy-related proteins, acting as transcriptional repressors [14]–[17]. Mammalian HES/HEY family proteins represent essential components to affect critical developmental decisions like nervous system, somitogenesis, blood vessel and heart development. Previous results suggested that HES genes play an essential role in the development of the nervous system, inactivation of HES1, HES3 and HES5 upregulate the expression of the proneural gene neurogenin 2 (Ngn2), deciding which cells choose non-neural versus neural cell fate [18]–[21]. In addition, HES7 controls the cyclic expression of lunatic fringe and is essential for coordinated somite segmentation [22]–[24]. The balance between HEY1 and HEYL expression regulates the differentiation of a subpopulation of TrkC (+) neurons in the dorsal root ganglia [25], [26]. HEY1 and HEY2 negatively regulate neuronal genes, promote maintenance of neural precursor cells, and increase late-born cell types in the developing brain [27]. It has also been suggested that HEY genes regulate arterial gene expression acting through the Shh-VEGF-Notch-HEY signaling pathway [28]–[31], restrict heart valve development and homeostasis through the Notch-HEY-Bmp2 regulatory axis [1], [3], [32]–[34]. HEY2 in mice and zebrafish can also regulate embryonic heart proliferative growth via opposing Gata5 or suppress atrial identity in the left ventricular compact myocardium by suppressing Tbx5 [32], [35], [36], whose upregulated expression may cause cardiac hypertrophy [37]–[39].

The HES proteins have a WRPW tetrapeptide, while a related YRPW peptide or a further degenerated YXXW were found in HEY proteins. The YXXW motif is followed by a conserved TE(I/V)GAF peptide with presently unknown function [17]. However, both DEC1 and DEC2 lack the WRPW/YRPW motif sequences and so far no evidence showed that their expressions were Notch-dependent. In addition, similar to the homologous genes in Drosophila, HES proteins are supposed to bind to both N-box and E-box DNA sequences (CACNAG, CANNTG). Nevertheless, HEY proteins preferentially bind to an E-box sequence [40]–[42]. HES genes play an essential role in the development of the nervous system, sensory organs, pancreas and endocrine cells, as well as lymphocytes. In contrast, HEY genes play critical roles in somitogenesis and the cardiovascular system [4], [8], [11], [16], [17], [43]. Although the structures of HES/HEY are conserved, they might have specific expression pattern and distinct roles [2], [12], [33], [37], [44], [45]. The known overlap in tissue and developmental stage suggested that there may be additional genetic interactions to be uncovered in compound HES and HEY deficient mutants [46]. Therefore, the elucidation of the classification, duplication and expression of the HES/HEY gene family will help to investigate the origination and diversity of their gene functions.

Based on evidences such as genome sizes and allozymes, Susumu Ohno suggested that the genomes of early vertebrates have been formed by two whole genome duplications (WGDs) [47]. In 1996, Sidow proposed that two large-scale gene duplications, most likely genome duplications, occurred in ancestors of vertebrates by using the phylogenetic analyses and sequence surveys of developmental regulator gene families [48]. Based on the “one-two-four” rule to explain the evolution of gene family in vertebrate, 2R model was proposed that an ancestral genome was duplicated to two copies after the first genome duplication (1R), and then to four copies after the second (2R) duplication [4], [49], [50]. More and more data suggested that an additional whole gene duplication occurred in teleost (3R or fish specific genome duplication, ruling “one-two-four-eight”) [51]–[55]. After WGDs, duplicated genes have experienced the gene loss and gain events during the vertebrate evolution [56], [57]. In 2010, Duncan studied the evolution of the Enhancer of split complex in Arthropods, which also included HES/HEY genes and mentioned one HES gene duplicated into 7 HES genes in fruit fly [58].

In this study, we made genome-wide identification of members of the HES/HEY gene family and analyzed their origin and evolution. Based on the analyses of the exon-intron structures and phylogenetic trees, we proposed a plausible scenario of the evolutionary history of the HES/HEY gene family. Meanwhile, we also verified the additional duplication of the HES/HEY genes in fishes and performed experiments to validate the differential expression of duplicated genes in zebrafish. The study of the origin, duplication and evolution of this gene family will help us to understand the function of them and provide clues for further functional verification.

## Results

### Genome Wide Identification Revealed the HES/HEY Genes in Metazoa

We performed a comprehensive search for genes of HES/HEY family in all kinds of species (see Methods). The results showed that this family exist in one kind of sponge (*Amphimedon queenslandica*), which is a primitive multicellular metazoan. No similar sequence of HES/HEY was found in plants, fungi and microbes by BLAST search at NCBI (see Methods). Local HMMER search results also indicated that no HES/HEY homogenous sequence was found in two protozoa (*Monosiga Brevicollis* and *Dictyostelium discoideum*). Thus, the HES/HEY family may originate in the common ancestor of Metazoa. HES/HEY contain two conserved domains: bHLH domain (Pfam: PF00010) and Orange domain (Pfam: PF07527). The bHLH domain was found in microbes, plants and animals, while the Orange domain only existed in Metazoa.

**Figure 1:**
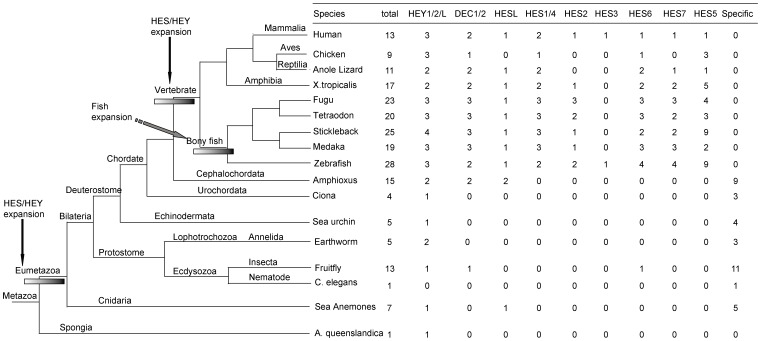
Two round expansions and a fish specific duplication of HES/HEY in all the species. The phylogenetic relationship of all the species investigated is in the left. The number of HES/HEY genes is in the right. Two round expansions and a fish specific duplication of HES/HEY are represented by arrows and bars.

We identified 216 HES/HEY genes in the 17 representative species, which belong to Porifera (sponge), Cnidaria (sea anemone), Ecdysozoa (*Caenorhabditis elegans* and fruit fly), Lophotrochozoa (earthworm), Echinodermata (sea urchin), Urochordata (ciona), Cephalochordata (amphioxus) and Vertebrata (others) (Figure 1, Table S1). We also found two potential HES/HEY sequences (ENSPMAP00000004014 and ENSPMAP00000001555) in the lamprey genome from Ensembl genome data. In sponge (*A. queenslandica*), there was only one HES/HEY sequence. In order to study the evolution of this family, we summarized the detailed information of HES/HEY genes in human, fruit fly and zebrafish in Table 1 and Table S2, S3. There were 13 HES/HEY genes in human and even 28 genes in zebrafish. There were 7 HES/HEY genes in the Cnidaria *Nematostella vectensis* and only one gene in *C. elegans*. Fruit fly contained 13 HES/HEY (1 HEY1/2/L, 11 HES, and one resembled DEC1/2). These fruit fly HES/HEY genes in our results were also studied and clustered with genes of other Arthropods in a previous study, which focused on the evolution of the Enhancer of split complex including several HES/HEY genes and other genes [58]. *Lumbricus rubellus* (Lophotrochozoa) had 5 HES/HEY (2 HEY1/2/L, 3 short sequences which may be incomplete sequences). *Strongylocentrotus purpuratus* (Echinodermata) contained 5 HES/HEY (1 HEY1/2/L and 4 HES), while *Ciona intestinalis* (Urochordata) contained 4 HES/HEY (1 HEY1/2/L and 3 HES).

**Table 1:** Human 13 HES/HEY genes.

Gene name	Official symbol	NCBI gene ID	Full name	Ensembl gene ID	Ensembl protein ID	Protein length	Location(chr:start-end:strand)
HEY1	HEY1	23462	HEY1 hairy/enhancer-of-split related with YRPW motif 1	ENSG00000164683	ENSP00000338272	308	Chr:8∶80676248:80680092:-1
HEY2	HEY2	23493	HEY2 hairy/enhancer-of-split related with YRPW motif 2	ENSG00000135547	ENSP00000357348	337	Chr:6∶126070726:126082415∶1
HEYL	HEYL	26508	HEYL hairy/enhancer-of-split related with YRPW motif-like	ENSG00000163909	ENSP00000361943	328	Chr:1∶40089825:40105617:-1
DEC1	BHLHE40	8553	BHLHE40 basic helix-loop-helix family, member e40	ENSG00000134107	ENSP00000256495	412	Chr:3∶5020801:5027008∶1
DEC2	BHLHE41	79365	BHLHE41 basic helix-loop-helix family, member e41	ENSG00000123095	ENSP00000242728	482	Chr:12∶26272959:26278060:-1
HESL	HELT	391723	HELT helt bHLH transcription factor	ENSG00000187821	ENSP00000426033	242	Chr:4∶185939995:185941950∶1
HES1	HES1	3280	HES1 hairy and enhancer of split 1	ENSG00000114315	ENSP00000232424	280	Chr:3∶193853934:193856521∶1
HES4	HES4	57801	HES4 hairy and enhancer of split 4	ENSG00000188290	ENSP00000304595	221	Chr:1∶934344:935491:-1
HES6	HES6	55502	HES6 hairy and enhancer of split 6	ENSG00000144485	ENSP00000272937	224	Chr:2∶239146908:239148765:-1
HES2	HES2	54626	HES2 hairy and enhancer of split 2	ENSG00000069812	ENSP00000367065	173	Chr:1∶6475292:6479979:-1
HES3	HES3	390992	HES3 hairy and enhancer of split 3	ENSG00000173673	ENSP00000367130	186	Chr:1∶6304252:6305638∶1
HES5	HES5	388585	HES5 hairy and enhancer of split 5	ENSG00000197921	ENSP00000367714	166	Chr:1∶2460184:2461684:-1
HES7	HES7	84667	HES7 hairy and enhancer of split 7	ENSG00000179111	ENSP00000314774	225	Chr:17∶8023908:8027410:-1

* Location is on the GRCh37.

### Phylogenetic Trees and Classification of HES/HEY Genes

**Figure 2:**
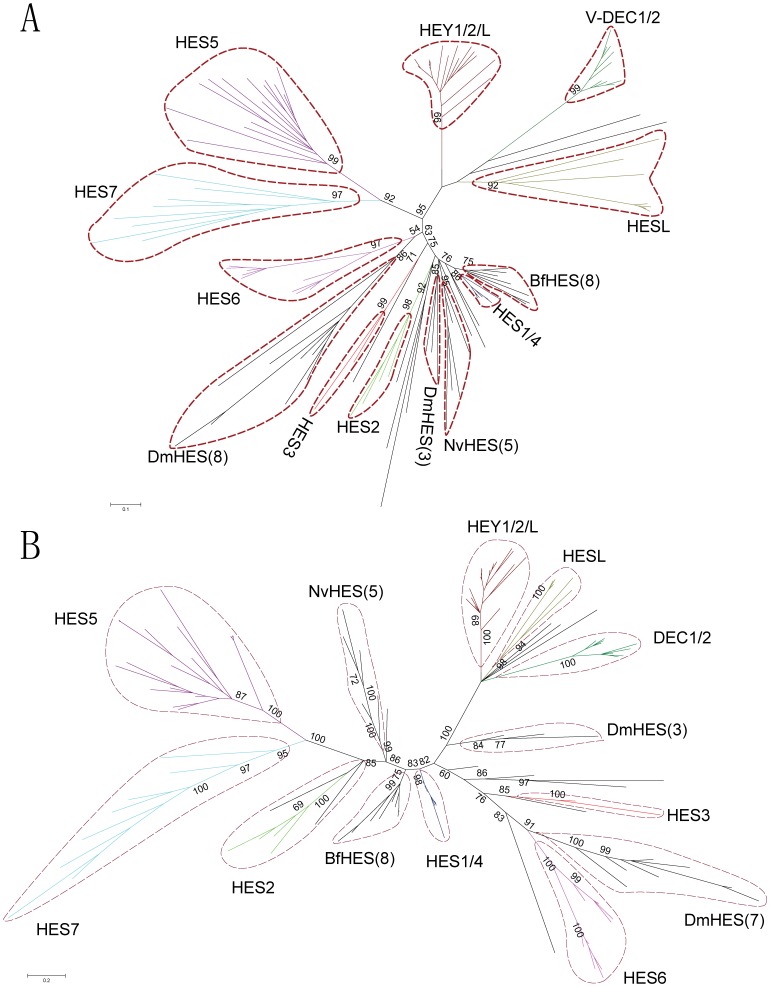
Unrooted Neighbor-Joining and Bayesian phylogenetic tree of HES/HEY genes. The trees were inferred by the Neighbor-Joining (NJ) with JTT model (A) and Bayesian (B) based on amino acid sequences of the bHLH and Orange domains. Colors denote different subgroups of HES/HEY, crimson: HEY1/2/L, dark green: V-DEC1/2, deep yellow: HESL, cyaneous: HES1/4, red: HES3, pink: HES6, prasinous: HES2, light blue: HES7, purple: HES5. Some species specific HES clusters including NvHES, DmHES, and BfHES were in black. Numbers in brackets refer to the numbers of the sequences in these species specific clusters. Nv, Dm and Bf are the abbreviations of *Nematostella vectensis, Drosophila melanogaster* and *Branchiostoma floridae.*

**Figure 3:**
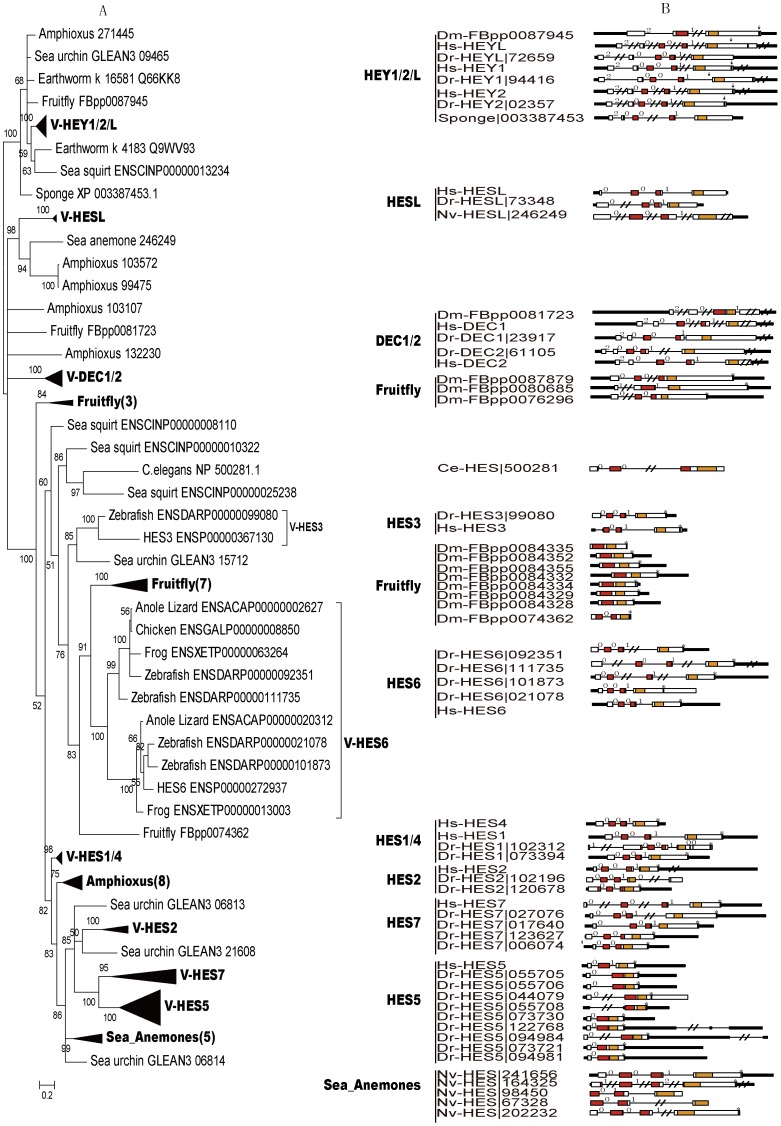
Phylogenetic tree and gene structure of HES/HEY genes. (A) Phylogenetic tree of HES/HEY genes were reconstructed by Mrbayes software. (B) The exon-intron structures of the HES/HEY genes in human, zebrafish, fruit fly, *C. elegans*, sea anemone and Sponge. Filled boxes: red for bHLH domain, orange for Orange domain, purple region with a star (*) on it represent the WRPW motif, blue region with an arrow (↓) on it represents YXXW motif; white boxes for other exon regions; and lines for introns. Numbers 0, 1, and 2: exon phases. The lengths of the boxes and lines are scaled based on the length of genes. The long introns, UTRs and exons were denoted by “//”. Numbers in the bracket of the compressed clades are the number of genes in those clades. Abbreviations of species names are in the following. Hs: *Homo sapiens*, Dr: *Danio rerio*, Dm: *Drosophila melanogaster,* Ce*: Caenorhabditis elegans*, Nv: *Nematostella vectensis.*

We performed multiple alignment for the amino acids sequences of bHLH and Orange domains in HES/HEY proteins (zebrafish HES/HEY as fish representative) (Figure S1). In order to view the overall topology of HES/HEY gene clusters, unrooted phylogenetics trees were constructed by Neighbor-jointing (NJ), Bayesian, Maximum-Evolution (ME), and Maximum-likelihood (ML) methods based on the multiple sequence alignment (Figure 2, 3, Figure S2, S3). The topologies of these trees were very similar except for the positions of HES3 clade, HES6 clade, and some species specific clades including sea anemone, fruit fly and amphioxus groups. Here we showed the unrooted NJ tree in Figure 2A and the Bayesian tree in Figure 2B to display the classification. The HES3 grouped with HES1/4 in NJ tree, while HES3 and HES6 clustered in one clade in the Bayesian tree. Based on the topology of phylogenetic tree, we classified them into four groups: HEY1/HEY 2/HEY L, DEC1/DEC2, HESL and HES1-7 (based on the human HES/HEY genes).

The partially expanded Bayesian phylogenetic tree was shown in Figure 3A to analyze the details of the classified clades. The full expanded subtrees of Bayesian method were in Figure S2 and the full trees constructed by ML, ME and NJ trees were shown in Figure S3A–C. Based on the expanded phylogenetic tree, we described the details of each groups by our classification in the following parts.

The first group is HEY1/HEY2/HEYL, which contained genes from all species except the *C. elegans* (Table 1). There are 3 members in most vertebrates and 1 member in most invertebrates. Genes in this group were clustered in a clade with the highest Bayesian posterior probability (100). The only one HEY gene in sponge was also located in this group, thus this group may be the oldest group and the ancestor of all other HES/HEY genes. The subtree of V-HEY1/2/L in Figure 3A is expanded in Figure S2. The high Bayesian posterior probabilities of three clades (V-HEY1 100, V-HEY2 100, and V-HEYL 98, respectively) suggest that they may diverge in the vertebrate lineage, but the exact timing is not clear. HEY1 and HEY2 all existed in zebrafish, frog, lizard, chicken and human.

The second group is the DEC1/DEC2 group, which includes DEC1 and DEC2 genes. They play pivotal functions in neurogenesis, neuroregulation, chondrogenesis, circadian rhythm, and are conserved in vertebrates. Here, we found that DEC1/2 existed in vertebrate, amphioxus, and fruit fly (Figure S2). However, we only found DEC1 in chicken, which suggest that the DEC2 may be lost or undetectable for the current uncompleted genome data. None of this group was found in ciona. The fruit fly (FBpp0081723) was considered as a member of this group for its exon-intron structure was similar to other genes in DEC1/DEC2, although it was not clustered with other DEC1/DEC2 genes in a clade by NJ and ME methods (Figure S3B–C). The third group is the HESL group, which was existed from *N. vectensis* to human. In phylogenetic trees, we noticed that this group was divided into two clades with high Bayesian posterior probabilities, which are vertebrate clade (100) and invertebrate clade (94). No HESL genes were identified in *C. elegans*, *S. purpuratus* and *C. intestinalis*.

The fourth group is HES1-7, which is the largest group among the four groups, with a high Bayesian posterior probability (100) in the base of this group. We noticed that the HES diverged into HES1-7 during the emergence of vertebrate, and formed many species specific clusters in vertebrate and invertebrate. The vertebrate HESs divided into HES1-7: HES6, HES1/HES4, HES2, HES3, HES5/7 five clades in phylogenetic trees (Figure 3A, Figure S2). The subgroup HES6 was divided into two clades in vertebrates except human. Only one sequence was found in human HES6, which maybe lost during the evolution. Most vertebrate HES1/4 clade had 2 members and only 2 sequences (human and zebrafish) existed in the HES3 clade (Figure 3A). For the HES2 and HES5/7 clades, more than 2 members in each of them were found in teleosts. For the invertebrate HES genes, some of them grouped with the corresponding vertebrate HES genes in a clade and some of them formed specific clusters. We described the species specific clades in the following.

### Species Specific Duplication of HES/HEY

We have noticed that species specific duplication of HES/HEY occurred in *N. vectensis*, *D. melanogaster*, *B. floridae*, teleosts, *X. tropicalis* and chicken. The *N. vectensis* cluster of 5 HESs represented the *N. vectensis* species specific duplication in the HES subgroup. Eight fly genes were grouped with V-HES6 (vertebrate-HES6) in one clade in Bayesian and ME tree (Figure 3A, Figure S3B), so we considered them as Fly-HES6 genes. We checked the chromosome location of these fruit fly HES6 genes, and found that 7 of them located in Chromosome 3R within 40 kb (Table S2), which may be the results of gene tandem duplications. One cluster of *B. floridae* HES (8) represented its specific duplication. The teleost specific duplication occurred mostly in HES5/7. The 5 *X. tropicalis* HES5 and 3 chicken HES5 scattered in the HES5 clade. This suggested that HES5 have more copies during the emergence of vertebrate, some of them have lost and some have duplicated in different lineages. We noticed that all the species specific duplications were occurred in HESs, not in HEY1/2/L, DEC1/2 and HESL clades.

### Gene Structure Analysis of HES/HEY Genes Indicates Exon/intron Loss Processes

In order to study the gene structure of HES/HEY genes, we analyzed the exon-intron structures of HES/HEY genes in some representative species based on the gene structure information from Ensembl or JGI (DOE, joint genome institute) (Figure 3B). Our results showed that genes in groups HEY1/HEY2/HEYL and DEC1/DEC2 had 5 exons and the same exon phases except for the two genes in fruit fly (FBpp0087945 and FBpp0081723), which have undergone different exon/intron loss and fusion processes. The exon phase of fruit fly|FBpp0081723 in HEY1/HEY2/HEYL was 2-0-1 compared to the 2-0-0-1 in other HEY1/HEY2/HEYL genes. All genes in HESL group had 4 exons and the same exon phases. These three groups diverged early in the Metazoa lineage, and two of them were existed in *N. vectensis*, a kind of Cnidaria. We noticed that the exon phase of the sponge HES/HEY gene (XP_003387453) was 2-0-0-1, which was similar to genes in the HEY1/HEY2/HEYL and DEC1/DEC2 groups.

The fourth group HES1-7 was involved in the exon fusion or intron loss in different subgroups. We noticed that the exon phases of human HES6, HES7, HES3, HES2 and HES1/4 genes were 0-0-1 and four exons, comparing with the 2-0-0-1 exon phase and five exons in HEY1/HEY2/HEYL groups (Figure 3B). The first exon of human HES3 was converted into 5′ UTR and the *C. elegans* sequence (NP_500281.1) lost the third intron, thus fused the third and fourth exons. We observed a diverse gene structure in the zebrafish HES2 subgroup, with an intron loss in ENSP00000020678 and an intron gain in ENSP00000002196. In subgroup HES5, genes had 2∼3 exons for the consequence of exon fusion. We found the different exon-intron structures for duplicated genes in fruit fly and zebrafish in some groups, which suggested these duplicated genes have undergone the exon fusion and intron loss processes after duplication. In HES super clade, fruit fly had 3 groups, one cluster with 7 genes, one cluster with 3 genes and one single copy (FBpp0074362). The cluster of 7 sequences (FBpp0084335 etc.) had only one exon, while FBpp0074362 had two exons. We considered these 8 sequences as Fly-HES6 because they were grouped in one clade in the ME tree (Figure S3B). The cluster of the 3 sequences (FBpp0087879 etc.) contained three exons and their exon phases were different. The exon-intron structure of 13 human HES/HEY shows that the HEY1/2/L and DEC1/2 retained the 5 exons with 2-0-0-1, while the HESL and HES1-7 lost the first exon (Figure S4). In order to describe the exon/intron loss processes more clearly, we summarized a list of the exon loss, exon fusion/intron loss in human, zebrafish and fruit fly compared with the sponge HEY (gi|340377873|) (Table S4).

### Functional Sites and Conserved Motifs in HES/HEY Proteins

It has been reported that the tetrapeptide WRPW or YRPW may be important to the HES/HEY function. We identified that tetrapeptide WRPW or YRPW was existed in most members of HES/HEY except DEC1/DEC2 and HESL groups. We noticed that a tetrapeptide FRPW existed in the last exon of Porifera *A. queenslandica*, which may be the ancestor of tetrapeptide WRPW or YRPW. Two teleost sequences (Fugu|ENSTRUP00000034668, Tetradon|ENSTNIP00000021570) conserved the most primitive tetrapeptide FRPW. In the HEY1/HEY2/HEYL genes, the tetrapeptide FRPW were mutated into YRPW. No tetrapeptide motif were found in HEY1/HEY2/HEYL of *N. vectensis* (Nemve1|28948|), suggesting it maybe incomplete sequence (only bHLH domain). In some members of fish HEY1/HEY2/HEYL, some genes lost the conserved tetrapeptide YRPW, for example, VLGW in Fugu|ENSTRUP00000007969, VQGW in Tetradon|ENSTNIP00000012093, AQGW in Stickleback| ENSGACP00000009942, GSGW in Medaba|ENSORLP00000017125, AQAW in Zebrafish|ENSDARP00000072659. In DEC1/DEC2 group and HESL group, all the members lost the conserved tetrapeptide. In group HES, the tetrapeptide YRPW were mutated to WRPW except some specific genes (C. elegans|NP_500281.1, Zebrafish|ENSDARP00000102196, Nemve1|98450, Nemve1|67328).

**Figure 4:**
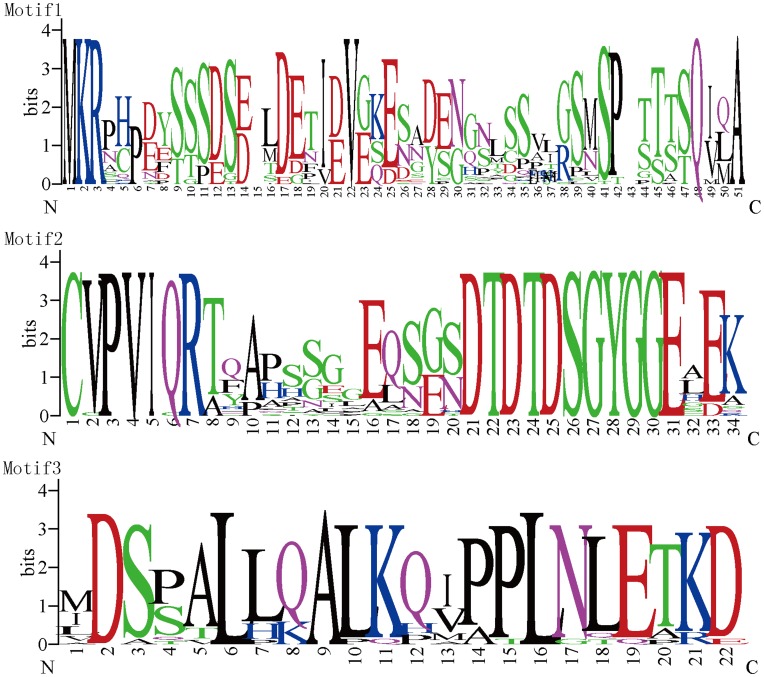
The sequence logos of three conserved motifs in subgroups of HES/HEY genes. The Motif 1 was created using an alignment of HEY1/2/L sequences near bHLH domain. The similar process was used to get Motif 2 (DEC1/2 sequence near Orange domain) and Motif 3 (DEC1 sequence in C-terminal). The overall height of each stack indicates the sequence conservation at that position, whereas the height of symbols within each stack reflects the relative frequency of the corresponding amino acid.

We found 3 motifs in several subgroups of HES/HEY (Figure 4). Most members in subgroup HEY1/HEY2/HEYL shared motif 1 located just N-terminal to the bHLH domain. Motif 2 was found in subgroup DEC1/DEC2 located at the C-terminal of the Orange domain. Almost all members of DEC1 proteins end with motif 3 in the C-terminal. Notably, HES/HEY protein sequences at the C-terminal of the Orange domain were proline-rich region. Although the biological functions of them are still unknown, the conserved amino acids among these three motifs implied that they might have similar functions.

### Teleost Specific Duplication of HES/HEY in Five Teleosts

Since much more HES/HEY genes were identified in the bony fishes, we further analyzed the HES/HEY genes in teleosts and constructed a Bayesian phylogenetic tree for human and fish HES/HEY genes (Figure S5). The HES/HEY gene expansion in teleosts may be the result of special whole genome duplication in fishes (3R WGD) [51]–[55]. We noticed that teleosts HEY/HES experienced species specific evolution in independently after they split from others. Even some teleosts formed their own specific HEY/HES gene clusters through gene tandem duplications, similar to the fruit fly or amphioxus specific clusters. Only in HESL, HEYL and HES3 clades, every teleost contains one or zero copy, while all of the other clades have more than one duplicated copies of teleosts. Zebrafish-specific HES5 cluster (5 sequences) and stickleback -specific HES5 cluster (9 sequences) were with high probability (100%) located in HES5 clade (Figure S5).

**Figure 5:**
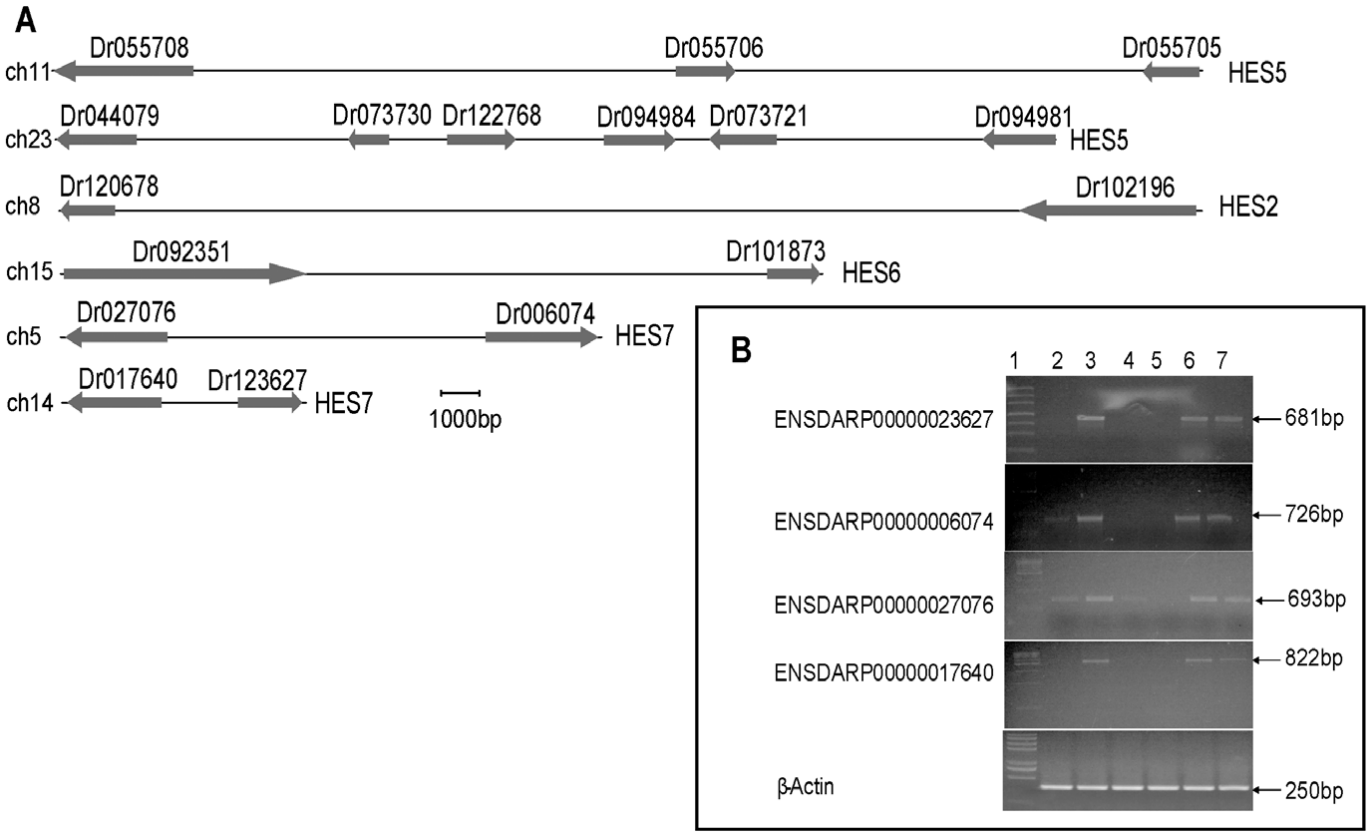
Zebrafish HES/HEY gene loci and expression pattern. (A) The gene loci of the zebrafish HES2, HES6, HES5 and HES7 genes. The arrows towards the right referred the gene on the positive-strand (+). The arrows towards the left referred the gene on the negative-strand (−). We cut short the Ensembl protein ID to reserve the last six digits in this diagram. For example, ENSDARP00000123627 is truncated as Dr123627. (B) Expression of HES7 genes in zebrafish. Transcripts of ENSDARP00000023627, ENSDARP00000006074, ENSDARP00000027076, ENSDARP00000017640 and β-actin were detected by RT-PCR. Lane 1 showed the molecular weight marker of 100 bp ladder. Gene expression patterns were obtained using total RNAs extracted from eye (lane 2), gill (lane 3), female ovary (lane 4), male testis (lanes 5), heart (lanes 6), and liver (lane 7). β-actin was performed to assess quantitative variations in mRNAs among all the samples.

In order to analyze the teleosts specific duplicated HES/HEY genes, we drew the graph displaying the gene loci of the zebrafish specific duplicated HES2, HES6, HES5 and HES7 genes (Figure 5A). We noticed that zebrafish HES5s were located on the two different chromosomes (ch11 and ch23), and 3 genes in ch11 and 6 genes in ch23 suggesting that they were expanded in tandem. This is similar to mouse SCD1/2/3/4 in Figure 4A of [59], which are confirmed by the authors to be expanded to four members. The two HES5 gene clusters (ch11 and ch23) correspond to the two zebrafish clusters in phylogenetic tree. Similarly, 4 HES7 genes were located on chr5 and chr14. And 2 HES2 and 2 HES6 genes were located in chr8 and chr15, respectively. Based on the above results, we inferred that 3R WGD and gene tandem duplications may be the reason that resulting in the expansion of these four HES genes.

**Figure 6:**
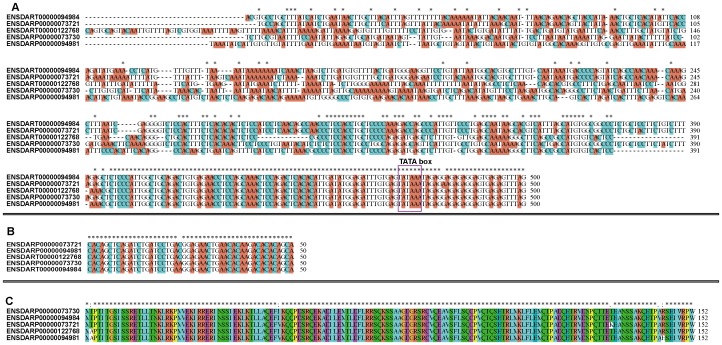
The alignment of the promoter, 5′ UTR and protein sequences in zebrafish HES5. (A) Multiple sequence alignment of the promoter sequences of zebrafish HES5 genes using the ClustalX2. The TATA box was circled by purple box. (B) Alignment of 5′ UTR sequence of zebrafish HES5 genes. (C) Alignment of zebrafish HES5 protein sequences.

To further study whether the zebrafish HES5 clusters were from tandem duplications, multiple alignments were conducted by ClustalW (Figure 6). The results showed that the downstream of the 5 promoters are conserved in many nucleotides, thus these regions may interact with TFs and other functional proteins (Figure 6A). The 5′ UTRs (Figure 6B) and protein sequences (Figure 6C) of the 5 zebrafish HES5 were almost identical except for several sites. The five HES5 genes (ENSDARP00000094981, ENSDARP00000073730, ENSDARP00000122768, ENSDARP00000094984, ENSDARP00000073721) were located on the same chromosome (chr23, Figure 5A) as neighbor genes. Considering the cluster loci and sequence similarity of these 5 genes, we inferred that they were expanded in tandem duplications.

### Expression Pattern of Zebrafish HES Genes

We next addressed the expression location of those duplicated zebrafish genes based on the information obtained from the ZFIN (Table S5, http://zfin.org/cgi-bin/webdriver?MIval=aa-ZDB_home.apg). Sheet 1 of Table S5 indicated the raw data of DEC1, DEC2, HEY1, HEY2, HEYL, HES1, HES3, HES5, HES6, HES7 expression pattern in different stages of zebrafish. We chose the tandem duplicated sequences (members of HES2, HES5, HES6, HES7) investigated from sheet 1, then summarized the gene information (name in zebrafish, Ensembl gene ID, Ensembl protein ID), expression stage range, expression anatomy of them in sheet 2 of Table S5. We noticed that HES7 (ENSDARP00000006074) was expressed in segmental plate and tail bud, whose stage range is 75%-epiboly to 10–13 somite stage (8 h–15 h). However, the homologous gene of HES7 (ENSDARP00000027076) was expressed in lateral mesoderm and mesoderm in the same stages. Although their amino acid and nucleotide sequences were highly similar, the expression patterns of them are diverse. We also checked the expression pattern (human body sites) of all 13 human HES/HEY genes based on the NCBI Unigene information (Table S6). The HEY1, DEC1 and HES1 expressed in most tissues investigated, while HESL, HES3 and HES7 expressed in fewer tissues.

Furthermore, we tested the expression of the duplicated genes in HES7 (Figure 5B), HES2 (Figure S6A) and HES6 (Figure S6B) by RT-PCR in adult zebrafish tissues including eye, gill, female ovary, male testis, heart, and liver. We found that the four members of HES7 (ENSDARP00000023627, ENSDARP00000006074, ENSDARP00000027076, and ENSDARP00000017640) show different expression patterns. All the four members are expressed in gill, heart and liver. ENSDARP00000006074 and ENSDARP00000027076 are also expressed in eye. Nevertheless, ENSDARP00000027076 is uniquely expressed in ovary (Figure 5B).

## Discussion

### Origin and Evolution of HES/HEY Genes

**Figure 7:**
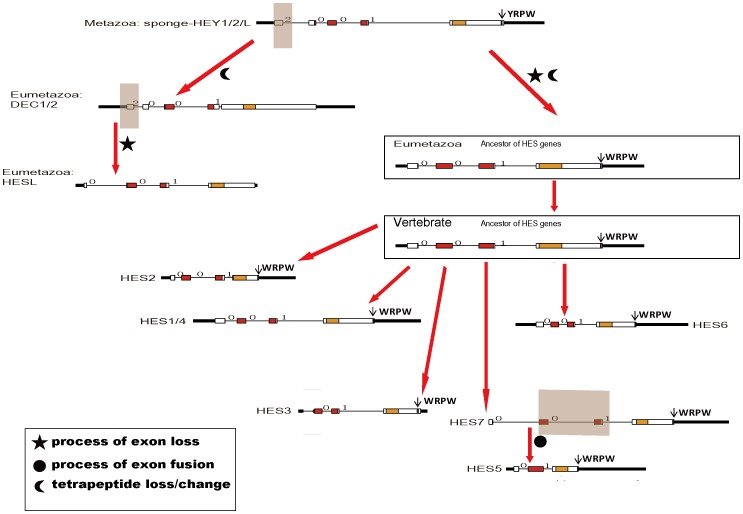
A model for the evolutionary process of the HES/HEY gene family. Filled boxes of the gene exon-intron structure: red represent bHLH domain, orange represent Orange domain, purple represent WRPW motif, blue represent YXXW motif; white boxes: other exon regions; lines: introns. Numbers 0, 1, and 2: exon phases. The length of the boxes and lines are scaled based on the length of genes. The transparent faint red boxes above exons or introns indicate the fused exons or lost exons.

Genes with bHLH domain are a kind of important transcription factor genes generally existing in both plants and animals [60]. We deduced that during the evolution, one of the bHLH domain containing genes obtained the Orange functional domain and thus the HES/HEY genes arose. Through our analysis, we confirmed that the HES/HEY genes only exist in Metazoa and none exist in other lineages such as protists, plants, bacteria, virus and fungi. In our analysis, sponge is the most primitive lineage in which we have found the the HES/HEY gene. The only one HES/HEY gene in sponge located on the HEY1/HEY2/HEYL group in the phylogenetic tree and its exon-intron structure was also similar to genes in this group of other species. This sequence has been mentioned as AmqbHLH1 in [61], and demonstrated that AmqbHLH1 has proneural activities in Xenopus and Drosophila. Thus, we inferred that the HEY1/HEY2/HEYL group is the ancestor group of HES/HEY transcription factor family. Based on the phylogenetic tree and exon-intron analysis, we proposed a model to infer the origin and evolution of HES/HEY genes, which were mainly involved in the processes of exon fusion and intron loss (Figure 7). Moreover, we concluded that two rounds expansions of HES/HEY occurred during the Metazoa and vertebrate emergence, and a third rounds duplication happened in teleosts (Figure 1).

The first expansion occurred during the emergence of the Metazoa (Figure 1). The ancestor of HES/HEY was found in one kind of sponge called *A. queenslandica*, which is a kind of Metazoa and appeared during the emergence of multicellular animal. The number of this family became 7 grouped in 3 (1 HEY1/HEY2/HEYL, 1 HESL, 5 HES) in *N. vectensis*, in contrast to 1 in sponge. We noticed the 5 HES in *N. vectensis* is specific cluster, the species specific clusters were also found in fruit fly and amphioxus. The second expansion occurred during the emergence of the vertebrates. The DEC1/DEC2 group was divided into two members, and this cluster contained the chordate amphioxus genes. Therefore, we suggested that in the ancestor of Eumetazoa, four members which is HEY, HES, DEC, HESL had existed. Some protostome lost one or more members in HES/HEY family, while vertebrate reserved the four members and expanded in some of them.

The exon-intron structure of the five species (human, zebrafish, sea anemones, fruit fly and *C. elegans*) revealed exon-intron loss occurred in the evolution of the HES/HEY gene family (Figure 3B). Genes in subgroups DEC1/DEC2 and HEY1/HEY2/HEYL had five exons and the exon phase was 2-0-0-1. Subgroups HESL, HES1/HES4 and HES6 lost the first exon and reserved four exons and the exon phase was 0-0-1. The HES2 genes had three to five exons and diverse exon phases. In subgroup of fruit fly specific cluster, the process of exon fusion event took place more clearly. It has been reported that the ancestral Eumetazoan genome must have intron-rich genes, in contrast to intron gains, some lineage appear to have experienced extensive intron loss, notably the fly, nematode, and sea squirt [62]. Our results support this thesis. Especially in the fruit fly special subgroup of HES, 7 of 8 genes had only one exon and they reside on the neighboring region of fruit fly Chromosome 3. The fruit fly special cluster may emerge via the tandem replication mechanism soon after the fruit fly was split from the other species. Based on the above evidences, we proposed that the members of HES/HEY tended to lose exons or lose introns and fuse exons during the evolutionary process. Specially, the fruit fly genes in DEC1/DEC2 group have gained an intron at the end of the sequence encoded Orange domain.

The HEY1/HEY2/HEYL genes first existed in Porifera at the early period and had identical five exons structure with a tetrapeptide FRPW existed in the last exon. In the HEY1/HEY2/HEYL genes, the tetrapeptide FRPW were mutated into YRPW. As the gene duplications and mutations occurred, the DEC1/DEC2 genes retained the main gene structure of the HEY1/HEY2/HEYL genes, however, lost the tetrapeptide YRPW. The HESL gene may emerge by losing the first exon of DEC1/DEC2 genes, so it does not have the tetrapeptide YRPW. In the HES group, which includes HES1-7, the tetrapeptide YRPW were mutated into WRPW. It has been reported that the Groucho/TLE proteins interacted with mammalian HES1 via its WRPW motif [14]. The HES1-7 genes in group 4 were duplicated and expanded several times during the evolution. They duplicated and formed species specific clusters in fruit fly and zebrafish. However, the tetrapeptide WRPW existed at the end of the protein sequences, which suggested that the tetrapeptide may be very critical for its function.

### Duplication of HES/HEY Genes

Several models for the gene duplication had been proposed, in some cases more than one model may be suitable to specific genes [63]. Except for the fruit fly cluster in subgroup of HES6, HES/HEY genes in the same group from different lineage tended to be clustered together in the phylogenetic tree, which indicated that they were duplicated before the divergence of the lineages. Genes clustered in subgroup HES6 fruit fly cluster were all from the fruit fly suggested that they duplicated after the divergence of the other lineages. But some genes from one organism were also massed into a cluster, such as the zebrafish HES5 genes, which suggested that these genes were duplicated recently.

In addition, there were 9 zebrafish HES/HEY genes clustered with human HES5 (ENSP00000367714), five of them highly resemble. It has been suggested that the increasing dosage of a particular gene was beneficial then a duplication of this gene may be fixed by positive selection [63]. We proposed that the zebrafish HES5 genes and the subgroup fruit fly genes duplicated and reserved for the benefit of increasing dosage. Fish-specific genome duplication occurred in teleosts. The 2R hypothesis assumed that 2 rounds of whole genome duplication (WGD) took place during the evolution of vertebrates. And there is evidence showing that fish have undergone a specific genome duplication [55]. Our results revealed the gene number of HES/HEY gene in the bony fish was significantly higher than that of other species (Figure 1). The gene numbers in subgroups DEC1/DEC2, HESL and HEY1/HEY2/HEYL in bony fish are comparable with those in non-fish vertebrates. On the contrary, the teleosts HES numbers are much more than non-fish vertebrates, exhibiting that they were expanded in HES2, HES5, HES6, and HES7. We performed the Wilcoxon Rank-Sum Test and the bony-fish genes almost doubled the number of non-fish vertebrates. These results also supported that another round of genome duplication occurred in bony fish. Hence, we suggested that the 3R WGD and gene tandem duplications contributed to the expansion of fish HES/HEY.

### Functions of HES/HEY

Fischer et al summarized the biological functions of HES or HEY gene [17]: Hes1 is important for the development of the nervous system, sensory organs (eye, inner ear), pancreas and endocrine cells, as well as lymphocytes; Hes7 plays critical roles in somitogenesis; Hey genes play essential roles in the cardiovascular system. In the absence of Hes1, Hes3 and Hes5, neural stem cells do not proliferate sufficiently but prematurely differentiate into neurons and become depleted without making the later born cell types such as astrocytes and ependymal cells [21], [64]. Dec1 and Dec2 can regulate the mammalian molecular clock through direct protein protein interactions with Bmal1 and/or competition for E-box elements [65]. How did HES/HEY family acquire these functions in evolution? Maybe the origin of the Notch signaling pathway can explain this: CSL-SuH (binding in promoter of some HES or HEY) is found in *Ustilago maydis* (Fungi) and *Monosiga brevicollis* (Choanoflagellata, more primitive than sponge), and the Lag1 and Beta-trefoil domains of CSL-SuH are specific to opisthokonts or even to metazoans [66]. Based on the evolution of Notch signal pathway, we inferred that the forming of CSL-SuH linked the transcription of HES/HEY with the Notch receptor and mediated the expression of HES/HEY [66].

The HES/HEY proteins were involved in regulating neurogenesis, vasculogenesis, mesoderm segmentation, myogenesis and embryonic development [14], [45]. Although the nervous system first existed in Cnidaria as nerve net. The emergence of the neuron-like cells should occur earlier and it was reported that the Sponge has a kind of cells resemble the neuron [61]. Thus, we suggested that the HES/HEY gene family may emerge at the same stage of the formation of nervous system. The HES/HEY genes promoted the simple nervous system to become diverged and more complicated.

## Conclusion

In this study, we identified all the members of the HES/HEY gene family in representative species. We found that this family was first generated during the emergence of Metazoa (in sponge), by means of obtaining the sequence encoding Orange domain which contains the sequence encoding the bHLH domain. Afterwards, the conserved tetrapeptide motif was obtained in the C-terminal of the proteins. The ancestor HES/HEY had 5 exons and its exon phase was 2-0-0-1 and then the other members were generated through exon fusion and intron loss. Species specific gene duplication and gene clusters were found in *N.vectensis, D.melanogaster*, and teleosts. We also studied the gene loci, expression patterns in zebrafish tissues (RT-PCR and ZFIN website). Overall, a model was proposed to show the HES/HEY origin and evolution.

## Materials and Methods

### Identification of HES/HEY Genes

The human HES/HEY gene list were obtained from the HES/HEY gene family [44]. The complete proteome data of human, chicken, green anole, *Xenopus tropicalis*, zebrafish, fugu, tetraodon, stickleback, medaka, *Ciona intestinalis*, fruit fly and *Caenorhabditis elegans* were downloaded from the Ensembl database (http://www.ensembl.org/). The complete proteome data of *Monosiga brevicollis*, amphioxus and sea anemone were downloaded from JGI (http://www.jgi.doe.gov/). The complete proteome of *Lumbricus rubellus* was downloaded from (http://salmo.bio.ed.ac.uk/genome_0_4/download.php) [67]. The complete proteome of *Strongylocentrotus purpuratus* was downloaded from (http://www.hgsc.bcm.tmc.edu/). Complete proteome of *Dictyostelium discoideum* was obtained from dictybase (http://www.dictybase.org). HMMER (http://hmmer.wustl.edu/) search using the Pfam profile PF00010 (bHLH domain) and PF07527 (Orange domain) against the above proteome sequences were performed and the results were manually refined to obtain the HES/HEY genes. BLAST search was then performed against the NCBI non-redundant protein sequences database and Ensembl database using the human 13 HES/HEY protein sequences to guarantee data integrity. HMMER search was performed in default parameter and BLAST search with an E value cutoff at 1e-30 because the bHLH domain and orange domain are very conserved and essential for HES/HEY proteins. The searching results were checked to reduce hits with partial bHLH and/or Orange domain and the other false positives. As the HMMER search using the Pfam profile PF07527, the human HES3 protein did not show significant Orange domain hit. We constructed a Hairy-Orange HMM model using the human and mouse Orange domain sequences, and performed HMMER search again. To remove the redundancy which may come from the JGI data, CD-HIT (http://weizhong-lab.ucsd.edu/cdhit_suite/cgi-bin/index.cgi?cmd=Server home) was used with default parameter [68]. We searched the HES/HEY by Blast in NCBI in lineages such as protist (http://www.ncbi.nlm.nih.gov/sutils/blast_table.cgi?taxid=Protozoa), plants (http://www.ncbi.nlm.nih.gov/mapview/static/MVPlantBlast.shtml?10), microbes (bacterial, archaeal, eukaryotic genomes) (http://www.ncbi.nlm.nih.gov/sutils/genom_table.cgi) and fungi (http://www.ncbi.nlm.nih.gov/sutils/genom_table.cgi?organism=fungi).

### Phylogenetic Analysis

For the phylogenetic analysis, the amino acid sequence of the most conserved bHLH and Orange domain. ClustalX (v2.0) [69] was used for the multiple sequence alignment of the jointed bHLH and Orange domains with default settings and the alignment results were manually refined. Nine HES/HEY genes were excluded in the phylogenetic analysis for their incomplete bHLH domains and/or Orange domains (Table S7). The phylogenetic trees were constructed using three different approaches: the Bayesian, the Maximum-Evolution (ME), the Maximum-likelihood (ML) methods and Neighbor-jointing (NJ). Bayesian analyses were performed in MrBayes 3.1.2 [70] with the mixed amino acid substitution model. Markov chains were run for 20,000,000 generations with sample frequency of 250. MEGA (v5.0) [71] (http://www.megasoftware.net/) was used to construct Minimum-Evolution (ME) phylogenetic tree with 1000 replicate interior-branch test, using the p-distance model and partial deletion (Gaps/Missing data treatment). The NJ trees were calculated using MEGA5 software [71] with the Jones-Taylor-Thornton (JTT) model. The confidence degree of nodes in NJ tree was assessed to carry out 1000 replicate interior-branch test. Gaps/Missing data treatment of NJ was partial deletion, too. The ML analysis was performed using PhyML3.0 [72]. The best fitting substitution models for our dataset were determined with the Akaike Information Criterion (AIC) using ProtTest 2.4 [73]. The AIC favored the JTT+I+G for the jointed amino acid sequences. The confidence degree of nodes in the phylogenetic trees was performed by bootstrapping with 100 replicates. The four analyses resulted in largely congruent tree topologies.

### Exon-intron Structure Analysis and Motif Search

For the exon-intron structure analysis, we chose five species among the main lineages of the Invertebrates and Vertebrates: human, zebrafish, fruit fly, *C. elegans* and sea anemone. The diagrams of exon-intron structure were painted using the R script (v2.12.0) (http://www.r-project.org/) based on the information obtained from the Ensembl and JGI. The locations of the domains were marked on the exons in different colors. Based on the complete protein sequence alignment conducted by ClustalX, several conserved motifs were retrieved manually. The sequence WebLogos of these motifs were produced by online server (http://weblogo.berkeley.edu/logo.cgi) based on the alignment results produced by ClustalW.

### RT-PCR Examination of HES/HEY Tissue Expression Patterns in Zebrafish

Breeding wild-type zebrafish (Danio rerio) (AB) were maintained and embryos were raised under standard library conditions [74]. Total RNA was extracted from tissues using the Trizol reagent (Invitrogen). RNA samples were digested with RNase Free DNase I (Promega). An aliquot of each RNA sample was used to spectrophotometrically (using a NanoDrop ND-1000; NanoDrop Technology, Wilmington, DE, USA) to determine the RNA quality (A260/A280>2.0) and concentration. First-strand cDNA was synthesized from total RNA with oligo (dT) priming using SuperScript II reverse transcriptase (Invitrogen) according to the manufacturer’s instructions. PCR products were analyzed on 2% agarose gel and sequenced. The primers used for the RT-PCR assays were in Table S8. β-actin was chosen as an internal control for normalization of the gene expression levels.

## Supporting Information

Figure S1 **Alignment for the phylogenetic analysis.**
(PDF)

Figure S2 **Expanded subtrees of** **Figure 3** **.**
(PDF)

Figure S3 **Phylogenetic trees by some other methods.** (A) Phylogenetic ML tree of HES/HEY genes (JTT+I+G). (B)Phylogenetic ME (JTT) tree of HES/HEY genes. (C) Phylogenetic NJ (JTT) tree of HES/HEY genes.
(PDF)

Figure S4 **Exon-intron structure of human HES/HEY genes.** The color representations have described in Figure 3. The four groups of HEY1/2/L, DEC1/2, HESL, and HES1-7 were circled by purple boxes.
(PDF)

Figure S5 **The Bayesian tree of HES/HEY genes in human and five representative species of teleost.**
(PDF)

Figure S6 **HES2 (a) and HES6 (b) expression in zebrafish tissues detected by RT-PCR.**
(PDF)

Table S1 **List of HES/HEY genes in the genomes investigated.**
(XLS)

Table S2 **13 HES/HEY genes in Fruit fly.**
(DOC)

Table S3 **28 HES/HEY genes in zebrafish (Zv9).**
(DOC)

Table S4 **The exon loss, exon fusion/intron loss of HES/HEY in human, zebrafish and fruit fly.** All the sequences were compared with the sponge HEY gi|340377873|ref|XP_003387453.1|, which is considered as the ancestor of HES/HEY.
(XLS)

Table S5 **The expression pattern of HES/HEY genes in zebrafish.**
(XLS)

Table S6 **The expression pattern of HES/HEY genes in human body sites.** Based on the information of Unigene in NCBI, the data of TPM (transcripts per million) was extracted. The “+” represents the value of TPM. One “+”: 0–50, two “+”: 50–100, three “+”: >100.
(XLS)

Table S7 **The excluded sequences in the phylogenetic analysis.**
(DOC)

Table S8 **The primers used for the RT-PCR assays.**
(DOC)
