# Supplementary material for: Comparative and Evolutionary Analysis of the HES/HEY Gene Family Reveal Exon/Intron Loss and Teleost Specific Duplication Events
Source: PLoS One. 2012 Jul 13;7(7):e40649. doi: 10.1371/journal.pone.0040649 (PMC3396596; doi:10.1371/journal.pone.0040649)

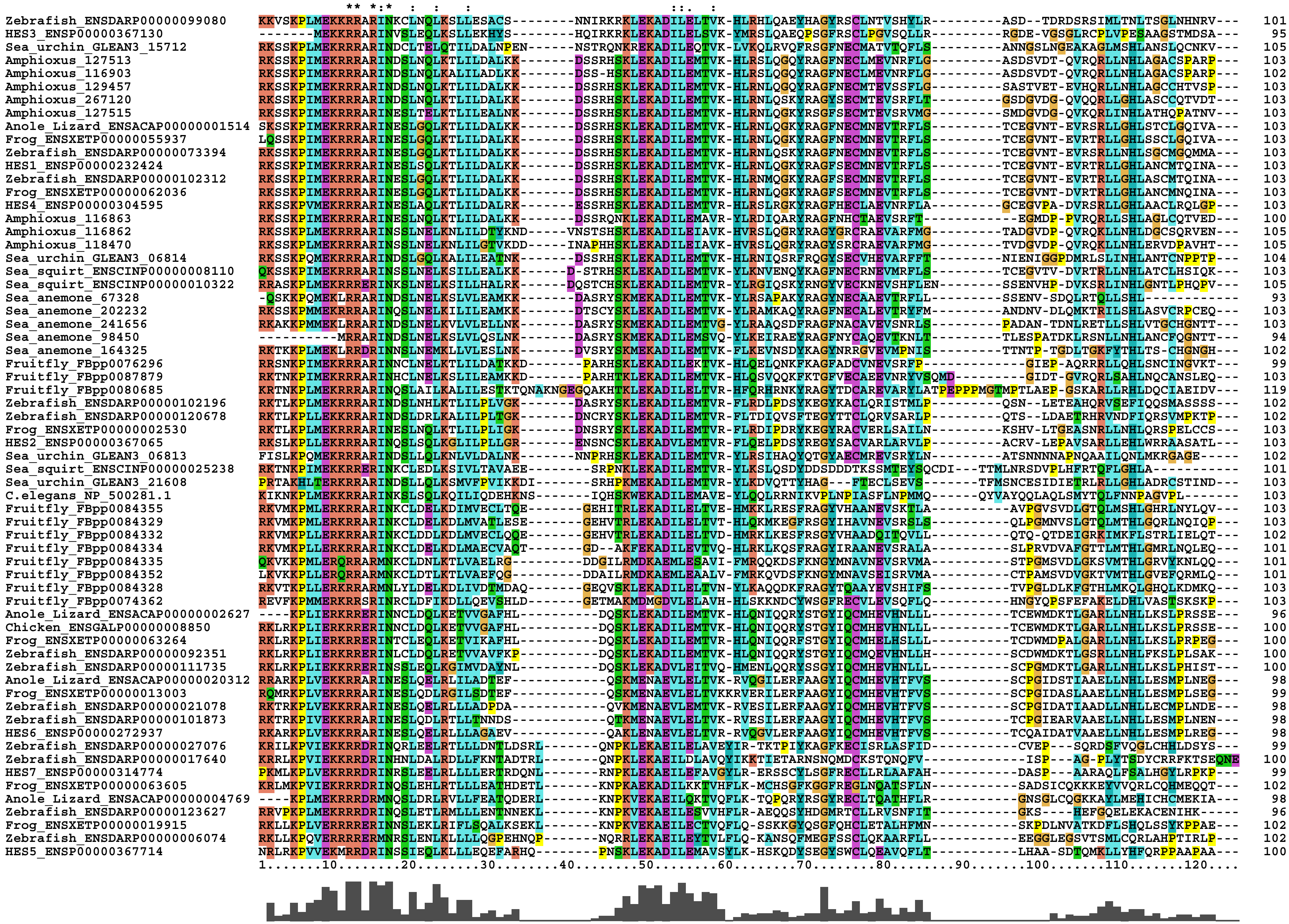
Zebrafish_ENSDARP00000099080 101
HES3_ENSP00000367130 95
Sea_urchin_GLEAN3_15712 105
Amphioxus_127513 103
Amphioxus_116903 102
Amphioxus_129457 103
Amphioxus_267120 103
Amphioxus_127515 103
Anole_Lizard_ENSACAP00000001514 103
Frog_ENSXETP00000055937 103
Zebrafish_ENSDARP00000073394 103
HES1_ENSP00000232424 103
Zebrafish_ENSDARP00000102312 103
Frog_ENSXETP00000062036 103
HES4_ENSP00000304595 103
Amphioxus_116863 100
Amphioxus_116862 105
Amphioxus_118470 105
Sea_urchin_GLEAN3_06814 104
Sea_squirt_ENSCINP00000008110 103
Sea_squirt_ENSCINP00000010322 105
Sea_anemone_67328 93
Sea_anemone_202232 103
Sea_anemone_241656 103
Sea_anemone_98450 94
Sea_anemone_164325 102
Fruitfly_FBpp0076296 99
Fruitfly_FBpp0087879 103
Fruitfly_FBpp0080685 119
Zebrafish_ENSDARP00000102196 102
Zebrafish_ENSDARP00000120678 102
Frog_ENSXETP00000002530 103
HES2_ENSP00000367065 103
Sea_urchin_GLEAN3_06813 102
Sea_squirt_ENSCINP00000025238 101
Sea_urchin_GLEAN3_21608 103
C.elegans_NP_500281.1 103
Fruitfly_FBpp0084355 103
Fruitfly_FBpp0084329 103
Fruitfly_FBpp0084332 102
Fruitfly_FBpp0084334 101
Fruitfly_FBpp0084335 101
Fruitfly_FBpp0084352 101
Fruitfly_FBpp0084328 103
Fruitfly_FBpp0074362 103
Anole_Lizard_ENSACAP00000002627 96
Chicken_ENSGALP00000008850 100
Frog_ENSXETP00000063264 100
Zebrafish_ENSDARP00000092351 100
Zebrafish_ENSDARP00000111735 100
Anole_Lizard_ENSACAP00000020312 98
Frog_ENSXETP00000013003 99
Zebrafish_ENSDARP00000021078 98
Zebrafish_ENSDARP00000101873 98
HES6_ENSP00000272937 98
Zebrafish_ENSDARP00000027076 99
Zebrafish_ENSDARP00000017640 100
HES7_ENSP00000314774 99
Frog_ENSXETP00000063605 102
Anole_Lizard_ENSACAP00000004769 98
Zebrafish_ENSDARP00000123627 96
Frog_ENSXETP00000019915 102
Zebrafish_ENSDARP00000006074 102
HES5_ENSP00000367714 100
1.......10.......20.......30.......40.......50.......60.......70.......80.......90.......100.......110.......120....

```
                                           ** *:*  :  :    :                        ::.   :
Chicken_ENSGALP00000001713      NRLRKPIVEKLRRDRINSSIEQLKLLLEKEFQRHQ--------PNSKLEKADILEMTVSYLK-YSRQDYCEGYAWCLKEALQFLS-----------LHSANTETQMKLICHFQRSQAMPKD---  101
Chicken_ENSGALP00000001706      NKLRKPVVEKMRRDRINSSIEQLKLLLEKEFQRHQ--------PNSKLEKADILEMAVSYLK-QQSFDFREGYSRCLQEAFHFLS-----------LHKVRTETQTKLLSHFQKSQSAAPE---  101
Chicken_ENSGALP00000001712      NKLRKPVVEKMRRDRINSSIEQLKLLLEKEFQRHQ--------PNSKLEKADVLEVAVSYLK-QQSQDFNSGYLRCLREAMHFLS-----------YYEPRKETQAQLIKHFCKAQIKQPA---  101
Anole_Lizard_ENSACAP00000003080 LQLRKPVVEKMRRDRINSSIEQLKLLLEKEFQQHQ--------PNSKLEKADILEMTVDYLK-QQSVDFKQGYSKCLHEAFQFMS-----------HHKVHAETQAKLVNHFQRSQLSPPD---  101
Frog_ENSXETP00000026362         NKLRKPIVEKMRRDRINNSIEQLKALLEKEFHKQE--------PNVKLEKADILEMAVSYLQ-QQKQDYKQGFSSCLREAVQFLC-----------YYPESGETQMKLLKHLQAPQKLSVA---  101
Frog_ENSXETP00000024266         NKIRKPMVEKMRRDRINSSINQLQNLLEKEFQLLQ--------PDSKPEKADILELAVKFLK-QQIQDFSQGYSNCLHETFAFLS-----------FHRTEEEMQLKLMNHFQCLDSQPRG---  101
Frog_ENSXETP00000061549         RQIRKPVVEKMRRDRINSSIKQLRMLLEKEFQRHQ--------PNSKLEKADILEMTVNYLK-EHQQDYNQGYSRCLEETLQFLS-----------HTEMQKPANLKLVQHFNRTVPADNN---  101
Frog_ENSXETP00000026359         NKLRKPVVEKMRRDRINSSIEQLKGLLETVFHKQQ--------PNVKLEKADILEMTVTYLR-QQTMDYKDGYSRCFEEVIDFLS-----------LHQKQPETA-KLISHFHSKATASSI---  100
Zebrafish_ENSDARP00000055705     HKLRKPVVEKMRRDRINNCIEQLKSMLEKEFQQQD--------PNAKLEKADILEMTVVFLK-QQLNAQIEGYSQCWRETISFLS-----------VGSEA-VAQRLQQEARRSAAPELTH---  100
Zebrafish_ENSDARP00000055706     HKLRKPVVEKMRRDRINNCIEQLKSMLEKEFQQQD--------PNAKLEKADILEMTVVFLK-QQLNAQIEGYSQCWRETISFLS-----------VGSEA-VAQRLQQEAQRSAAPELTH---  100
Zebrafish_ENSDARP00000044079     IKLRKPIVEKMRRDRINTCIDQLKSLLEKEFHSHD--------PSTKLEKADILEMTVSFLK-QQIRDFNEGYSHCWRESVHFLS-----------LHSNAGELQHLHSGPKTNSTMGSTP---  101
Frog_ENSXETP00000048128         RKIRKPVVEKMRRDRINSSIEQLRMLLEKEFEKHN--------LPSKPEKADILEVAVGFLQ-QHIKSYKEGYSKCVEDSVHFLS-----------AHNQH--SQGNLLRSFHGHQFSSGE---   99
Zebrafish_ENSDARP00000055708     ----------MRRDRINKCIEQLKILLKTEIKASQ--------PCSKLEKADILEMAVIYLK-NTAQSYADGYSRCIEETARFLS-----------AHKQTQKHSKPVDSCQITSEIAKHG---   91
Zebrafish_ENSDARP00000073721     NKLRKPMVEKIRRERINSSIEKLKTLLAQEFVKQQ--------PDSRQEKADILEMTLDFLR-RSQSAAGDGRSRCVQEAVSFLS-----------QCPVQTQSHTRLMKLFLHMQTPADQ---  101
Zebrafish_ENSDARP00000094981     NKLRKPMVEKIRRERINSSIEKLKTLLAQEFVKQQ--------PDSRQEKADILEMTLDFLR-RSQSAAGDGRSRCVQEAVSFLS-----------QCPVQTQSHTRLMKLFLHMQTPADQ---  101
Zebrafish_ENSDARP00000073730     NKLRKPMVEKIRRERINSSIEKLKTLLAQEFIKQQ--------PDSRQEKADILEMTLDFLR-RSQSAAGDGRSRCVQEAVSFLS-----------QCPVQTQSHTRLMKLFLHMQTPADQ---  101
Zebrafish_ENSDARP00000094985     NKLRKPMVEKIRRERINSSIEKLKTLLAQEFIKQQ--------PDSRQEKADILEMTLDFLR-RSQSAAGDGRSRCVQEAVSFLS-----------QCPVQTQSHTRLMKLFLHMQTPADQ---  101
Zebrafish_ENSDARP00000007386     NKLRKPMVEKIRRERINSSIEKLKTLLAQEFIKQQ--------PDSRQEKADILEMTLDFLR-RSQSAAGDGRSRCVQEAVSFLS-----------QCPVQTQSHTRLMKLFLHMQTPADQ---  101
Amphioxus_271445                RKKRRGIIEKRRRDRINNCLAELRRLVPTAFEK---------QGSAKLEKAEILQMTVD-YLKMLADYRGIGFRECANEVARYMVTIEGLDI--------QDPLRIRLMNHLQCIAAQREA---  103
Sea_urchin_GLEAN3_09465         RKKRRGIIEKRRRDRINNSLTELRRLVPAAFEK---------QGSAKLEKAEILQMTVD-HLKYLHDYRVLGFRECASEVARYLVTVEGMDI--------QDPLRLRLMSHLQCYVAQREF---  103
Earthworm_k_16581_Q66KK8        RKKRRGVIEKRRRDRINNSLSELRRLVPTAFEK---------QGSAKLEKAEILQMTVD-HLKLVQEYRMAGFRECAAEVARYLVSIEGMDL--------QDPLRLRLLSHLQCYCAQREA---  103
Fruitfly_FBpp0087945            RKKRRGVIEKKRRDRINSSLTELKRLVPSAYEK---------QGSAKLEKAEILQLTVE-HLKSLQDYHIIGFRECAAEVARYLVTIEGMDI--------QDPLRLRLMSHLQYFVQQREL---  103
Sponge_XP_003387453.1           RKKRRGIIEKRRRDRINNCLMELRRLVPAAFEK---------QGSAKLEKAEILQMTVD-HLRHLHDYRVMGFRECVAEVARYMTNVEGLDM--------KDPMRVRVLNHLENYLTQREL---  103
Zebrafish_ENSDARP00000002357     RKKRRGIIEKRRRDRINNSLSELRRLVPTAFEK---------QGSAKLEKAEILQMTVD-HLKMLQDFLSIGFRECLTEVARYLSSVEGLDS--------SDPLRVRLVSHLSSCASQREA---  103
Frog_ENSXETP00000047237         RKKRRGIIEKRRRDRINNSLSELRRLVPTAFEK---------QGSAKLEKAEILQMTVD-HLKMLQDFMSIGFRECLTEVARYLGSVEGLDS--------SDPLRVRLVSHLSSCASQREA---  103
HEY2_ENSP0000357348             RKKRRGIIEKRRRDRINNSLSELRRLVPTAFEK---------QGSAKLEKAEILQMTVD-HLKMLQDFMSIGFRECLTEVARYLSSVEGLDS--------SDPLRVRLVSHLSTCATQREA---  103
Anole_Lizard_ENSACAP00000001749 RKKRRGIIEKRRRDRINNSLSELRRLVPTAFEK---------QGSAKLEKAEILQMTVD-HLKMLQDFISIGFRECLTEVARYLTSVEGLES--------SDPLRVRLVSHLSTCASQREA---  103
Chicken_ENSGALP0000023905       RKKRRGIIEKRRRDRINNSLSELRRLVPTAFEK---------QGSAKLEKAEILQMTVD-HLKMLQDFMSIGFRECLTEVARYLTSVEGLDT--------SDPLRVRLVSHLSTCASQREA---  103
Chicken_ENSGALP0000025335       RKRRRGIIEKRRRDRINNSLSELRRLVPSAFEK---------QGSAKLEKAEILQMTVD-HLKMLHDYRSLGFRECLAEVARYLSIIEGLDA--------SDPLRVRLVSHLNNYASQREA---  103
HEY1_ENSP0000338272             RKRRRGIIEKRRRDRINNSLSELRRLVPSAFEKQVM-----EQGSAKLEKAEILQMTVD-HLKMLHDYRSLGFRECLAEVARYLSIIEGLDA--------SDPLRVRLVSHLNNYASQREA---  107
Anole_Lizard_ENSACAP00000013195 RKRRRGIIEKRRRDRINNSLSELRRLVPSAFEKQVIFFR-GVEGSAKLEKAEILQMTVD-HLKMLHDYRSLGFRECLAEVARYLSIIEGLDT--------ADPLRVRLVSHLNNYASQREA---  111
Frog_ENSXETP0000017194          RKRRRGIIEKRRRDRINNSLSELRRLVPSAFEKQVIQ-----QGSAKLEKAEILQMTVD-HLKMLHDYRSLGFRECLAEVARYLSIIEGMDT--------TDPLRVRLVSHLNNYASQREA---  107
Zebrafish_ENSDARP0000094416      RKRRRGIIEKRRRDRINNSLSELRRLVPSAFEK---------QGSAKLEKAEILQMTVD-HLKMLHDYRGLGFRECLAETARYLSIIEGLDN--------TDPLRIRLVSHLNSYASQREA---  103
HEYL_ENSP0000361943             RKKHRGIIEKRRRDRINSSLSELRRLVPTAFEK---------QGSSKLEKAEVLQMTVD-HLKMLHDFRSIGFRECLTEVIRYLGVLEGPSSR-------ADPVRIRLLSHLNSYAAEMEP---  104
Zebrafish_ENSDARP0000072659      RKKRRGIIEKRRRDRINHSLSELRRLVPSAFEK---------QGSSKLEKAEILQMTVD-HLKLLHDYRTLGFRECVGEVVRYLSSLEGVES--------SDPIGARLVSHLSHCASELDP---  103
Earthworm_k_4183_Q9WV93         FKLLPAIIEKRRRDRINYSLAELKRLVPAAFEK---------QGSSKLEKAEILKMTVD-HLKMMQDFRSVGFRECAAEVARYLVAIEGLDS--------HDPLCVRLLSHLHYYPSTAPT---  103
Sea_squirt_ENSCINP00000013234   RKKRRGVIEKRRRDRINNSLSELRRMIPAASEK---------HGSSKLEKAEILQLTVE-HLKTLQDYHSFGFRECVSEVARYMISMDGIQS----------SVRENLLSHLQNVMTHKPT---  101
Amphioxus_99475                 EISSHKIVERRRRHRINTCIAQLSQAIPAAFSKSVNRR---RGLSGKLEKAEVLEMAVS-YVKHIQRHFEEGYRECVKEVARYLAEVEGMNP--------QDVRFLRLLCHLQTRGPAARG---  109
Amphioxus_103572                EISSHKIVERRRRHRINTCIAQLSQAIPAAFSKSVNRR---RGLSGKLEKAEVLEMAVS-YVKHIQRHFEEGYRECVKEVARYLAEVEGMNP--------QDVRFLRLLCHLQTRGPAARG---  109
Sea_anemone_246249              SECSHKVIEKRRRDRINSCLSELAQLIPSAQ----NGK---QG-SGKLEKAEILELTVE-YVKKNLRKYWMGYSDCTAEVLRFLVAVEAMDP--------QQPCFQRLMAHLRHRHNLMVE---  104
Anole_Lizard_ENSACAP00000002279 IPVSHKVIEKRRRDRINRCLNELGKTVPMALAK---------QSSGKLEKAEILEMTVQ-YLRALHNYFHYGYHECMKNLVHYLTTVEQMET--------KDTKYARILAFLQSKARFVTE---  103
HESL_ENSP0000426033             TPVSHKVIEKRRRDRINRCLNELGKTVPMALAK---------QSSGKLEKAEILEMTVQ-YLRALHNYFHYGYHECMKNLVHYLTTVERMET--------KDTKYARILAFLQSKARLGAE---  103
Zebrafish_ENSDARP00000073348     TPVSHKVIEKRRRDRINRCLNELGKTVPMALAK---------QNSGKLEKAEILEMTVQ-YLRALHNYFHYGYHECMKNLVHYLTTVERMET--------KDTKYARILAFLQSKVVTEPV---  103
Frog_ENSXETP00000062321         APVSHKVIEKRRRDRINRCLSELGKTVPMALAK---------QNSGKLEKAEILEMTVQ-YLRALHNYFHYGYHECMKNLVHYLTTVERMET--------KDNKYARIVAFLQSKAHLSTE---  103
Amphioxus_103107                QRDLHRIVEKRRRDRINDCLANIRELLPEELVRQ--------KSCG---KAEILELTLM-HMKHLQGDFLAGYRECLGEAIRYMS---QSPV--------DGVSCEKIESHLRRHCQRLSP---   98
Fruitfly_FBpp0081723            DPLSHRIIEKRRRDRMNSCLADLSRLIPPQYQR---------KGRGRIEKTEIIEMAIR-HLKHLQSDYRSGYMDCMKEAAKFLY---DVHM--------QDF-CHRLLGRLQEHIDEMFK---   99
Amphioxus_132230                EPTPHKVIEKRRRDRINQRFEELRGLVPTARNSQ--------GVKN--DKVDLLHMTIE-HLKTLTDAVLAGFSECAYEALQYLLHEEHMPE--------DSELVVSLRSHLRNSQMSLDV---  102
Anole_Lizard_ENSACAP00000012353 YKLPHRLIEKKRRDRINECIAQLKDLLP-EHLKL--------TTLGHLEKAVVLELTLK-HVKALTEVFRSGFQMCAKEVLQYVAKHENA----------KELKSSQLISHLHRVASEVLQ---  101
Chicken_ENSGALP00000013509      YKLPHRLIEKKRRDRINECIAQLKDLLP-EHLKL--------TTLGHLEKAVVLELTLK-HVKALTEMFRSGFQLCAKELLQYVAKHDN-----------KELKAAQLVGHLHRVAAELLP---  100
DEC1_ENSP0000256495             YKLPHRLIEKKRRDRINECIAQLKDLLP-EHLKL--------TTLGHLEKAVVLELTLK-HVKALTEMFCSGFQTCAREVLQYLAKHENT----------RDLKSSQLVTHLHRVVSELLQ---  101
Zebrafish_ENSDARP00000023917     YKLPHRLIEKKRRDRINECIAQLKDLLP-EHLKL--------TTLGHLEKAVVLELTLK-HVKALNEMFRSGFHLCAKEVLQFLANQETM----------RDLTTAHIIEHLQKVASELIQ---  101
Frog_ENSXETP00000011372         YKLPHRLIEKKRRDRINECIAQLKDLLP-EHLKL--------TTLGHLEKAVVLELTLK-HVRSLSEMFHSGFQLCAEEALRFLQGGER----------------KELVAHLHRVASKLGP---   95
DEC2_ENSP0000242728             YKLPHRLIEKKRRDRINECIAQLKDLLP-EHLKL--------TTLGHLEKAVVLELTLK-HLKALTDAFHSGFQTCAKEVLQYLSRFESWTP--------REPRCVQLINHLHAVATQFLP---  103
Anole_Lizard_ENSACAP00000000427 YKLPHRLIEKKRRDRINECIAQLKDLLP-EHLKL--------TTLGHLEKAVVLELTLK-HLKALTDAFHSGFQTCTKEVLQYLSRFESWAA--------REQRCAQLIGHLHAVCARFLP---  103
Frog_ENSXETP00000050690         YKLPHRLIEKKRRDRINECIAQLKDLLP-EHLKL--------TTLGHLEKAVVLELTLK-HLKGLTDAFHSGFQTCAKEVLQYLSRFESWTS--------RDQRCTQLLNHLHTVSSQILS---  103
Zebrafish_ENSDARP00000061105     YKLPHRLIEKKRRDRINECIGQLKDLLP-EHLKL--------TTLGHLEKAVVLELTLK-HLNALTDAFHSGFQACAKEVLQYLNKVENWTA--------REQRCTRLINHLHKVSAQFQP---  103
                                1.......10........20........30........40........50........60........70........80........90.......100.......110.......120....
```

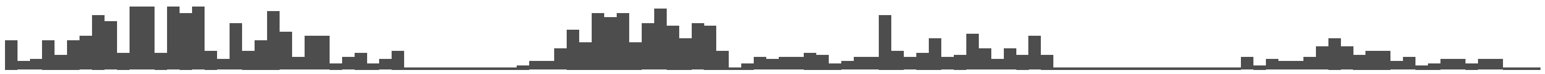

Supplement: Figure S1 — Alignment for the phylogenetic analysis. (PDF) [file pone.0040649.s001.pdf]
