# Supplementary figures and images for: Comparative and Evolutionary Analysis of the HES/HEY Gene Family Reveal Exon/Intron Loss and Teleost Specific Duplication Events
Source: PLoS One. 2012 Jul 13;7(7):e40649. doi: 10.1371/journal.pone.0040649 (PMC3396596; doi:10.1371/journal.pone.0040649)

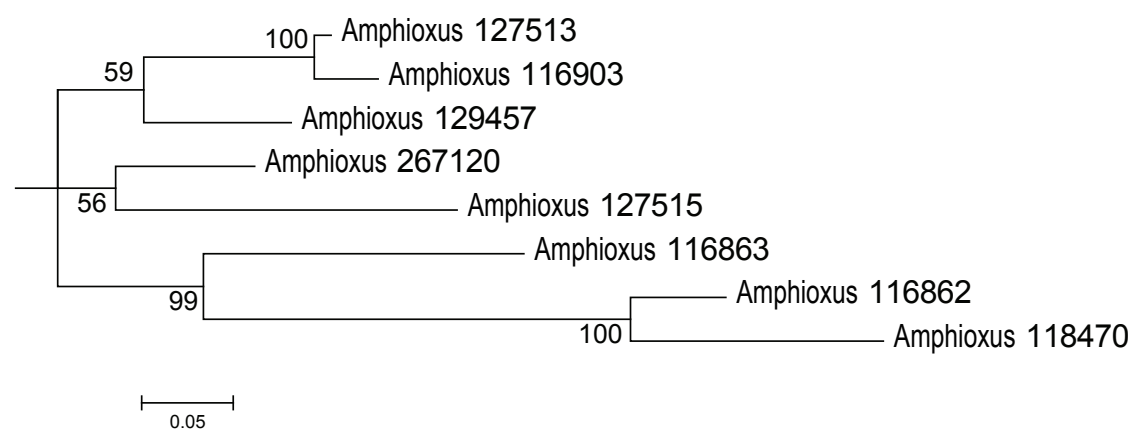

**Amphioxus (8)**

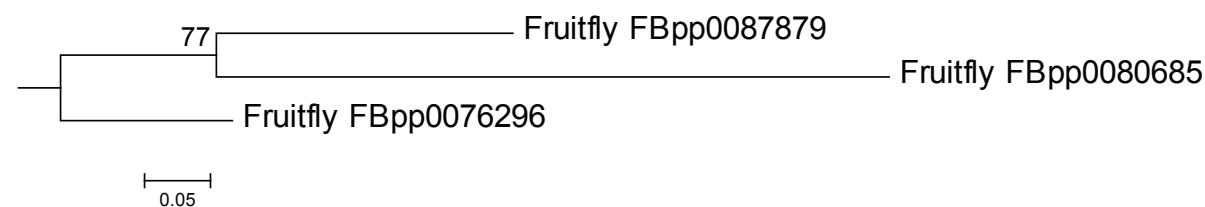

**Fruitfly (3)**

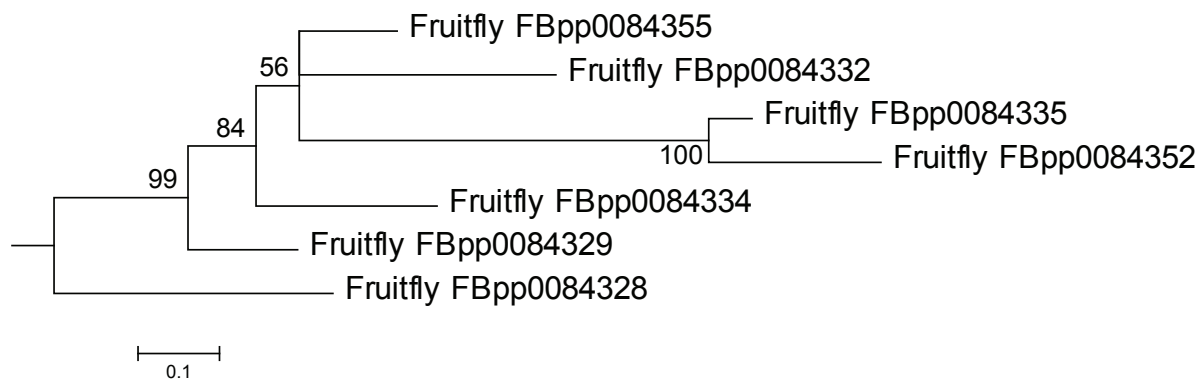

**Fruitfly (7)**

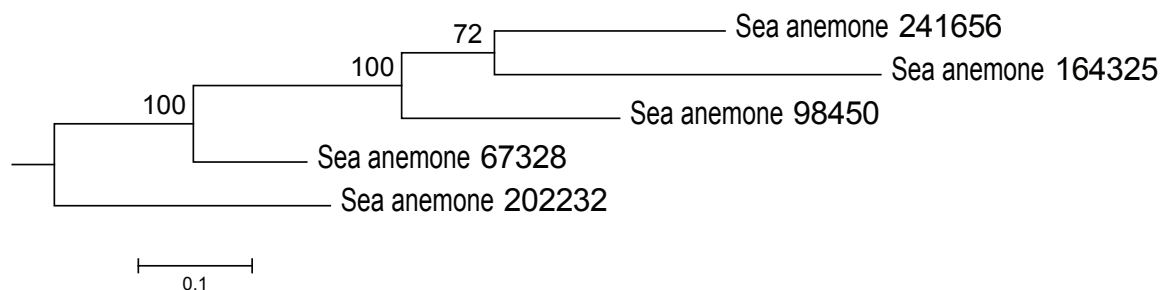

**Sea\_Anemones (5)**

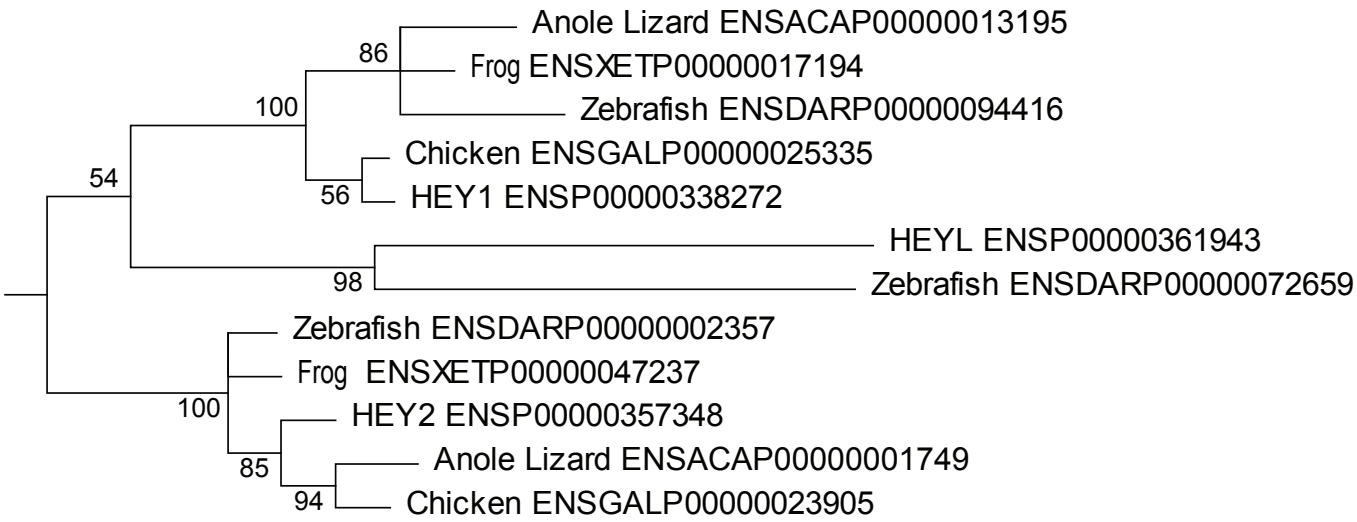

V-HEY1/2/L

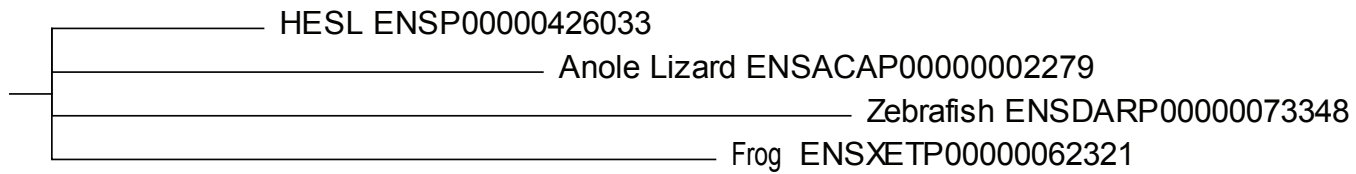

V-HESL

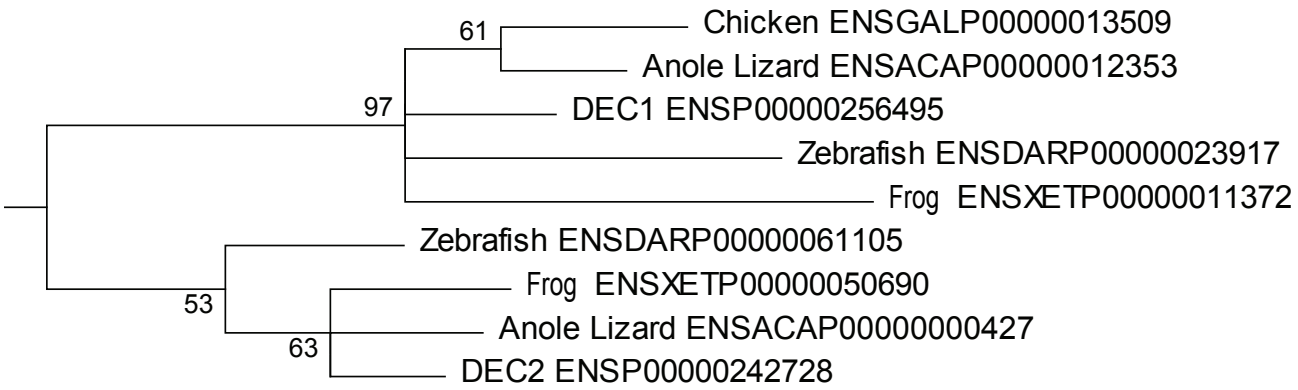

V-DEC1/2

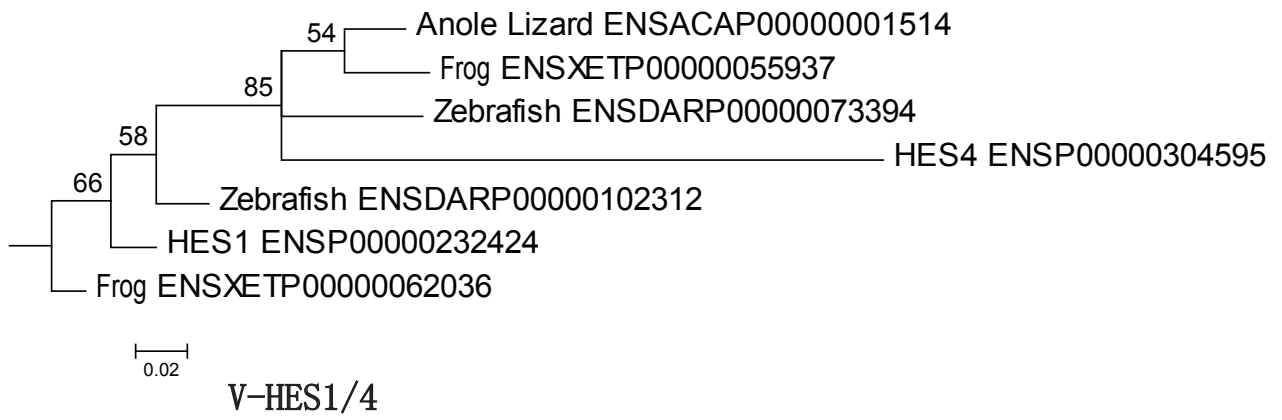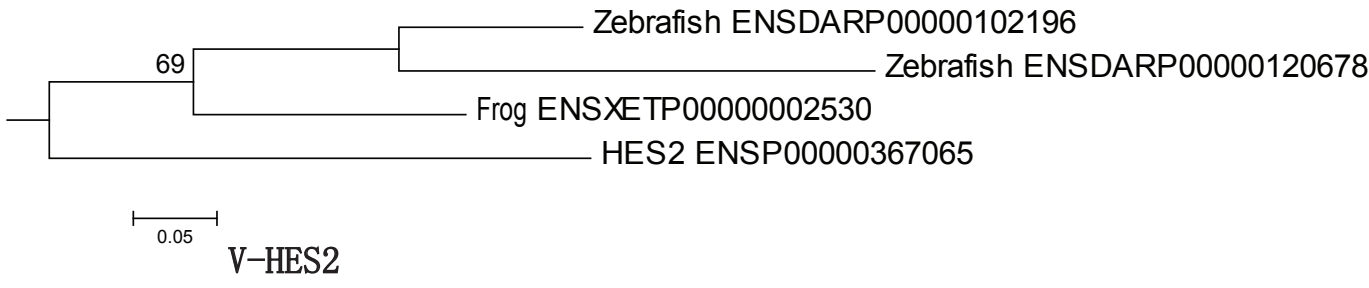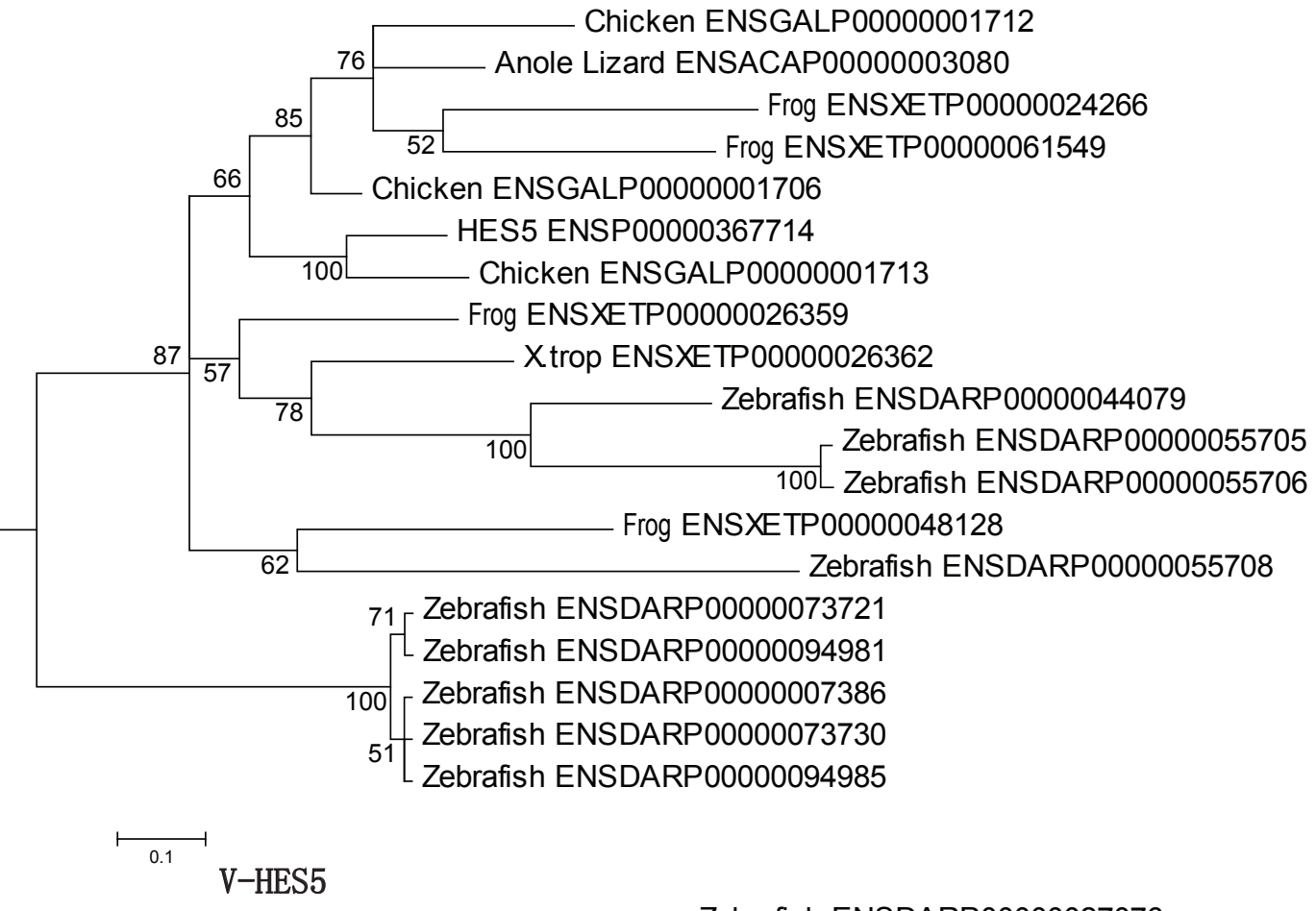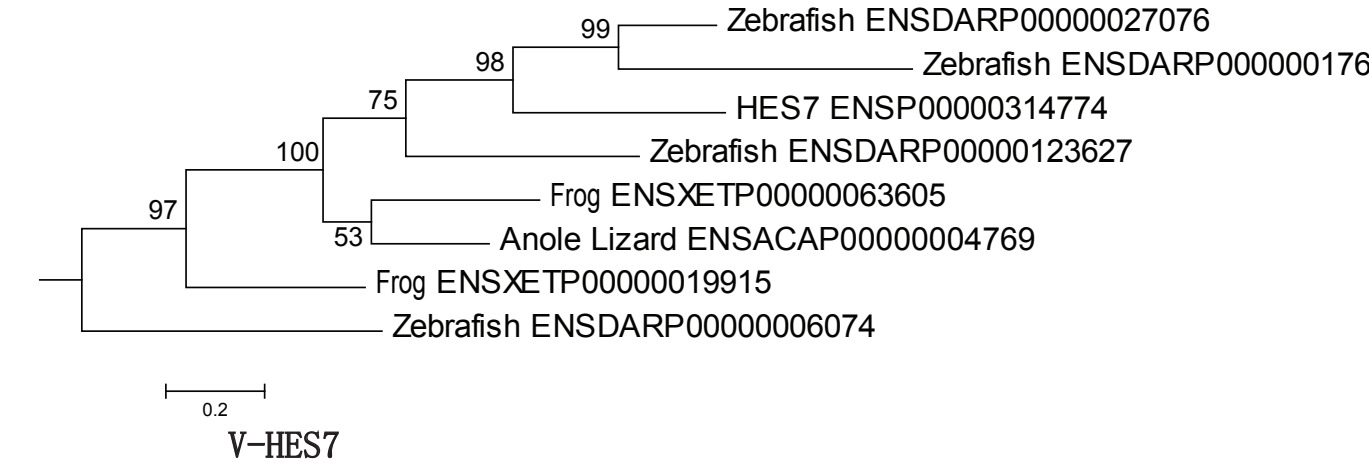

Supplement: Figure S2 — Expanded subtrees of Figure 3 . (PDF) [file pone.0040649.s002.pdf]

The Bayesian tree of HES/HEY genes in human and five Teleost

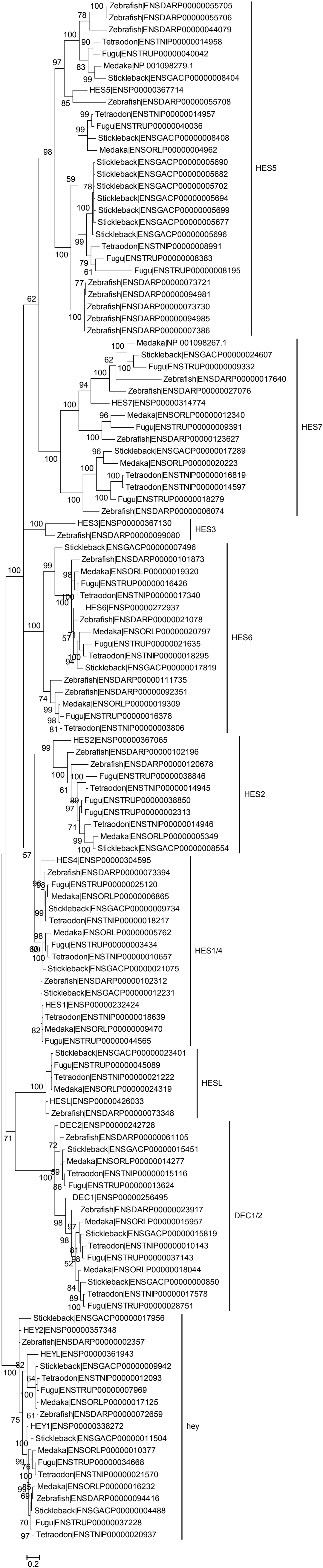

Supplement: Figure S5 — The Bayesian tree of HES/HEY genes in human and five representative species of teleost. (PDF) [file pone.0040649.s005.pdf]

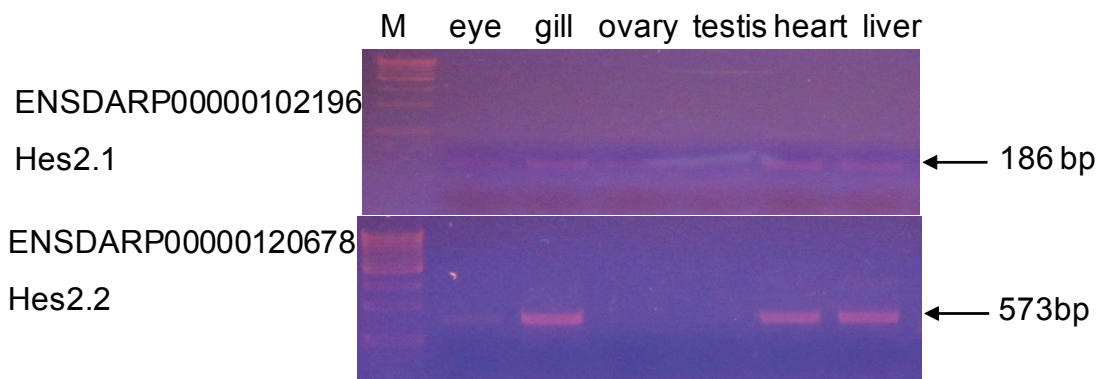

## HES2 expression in zebrafish by RT-PCR

(a)

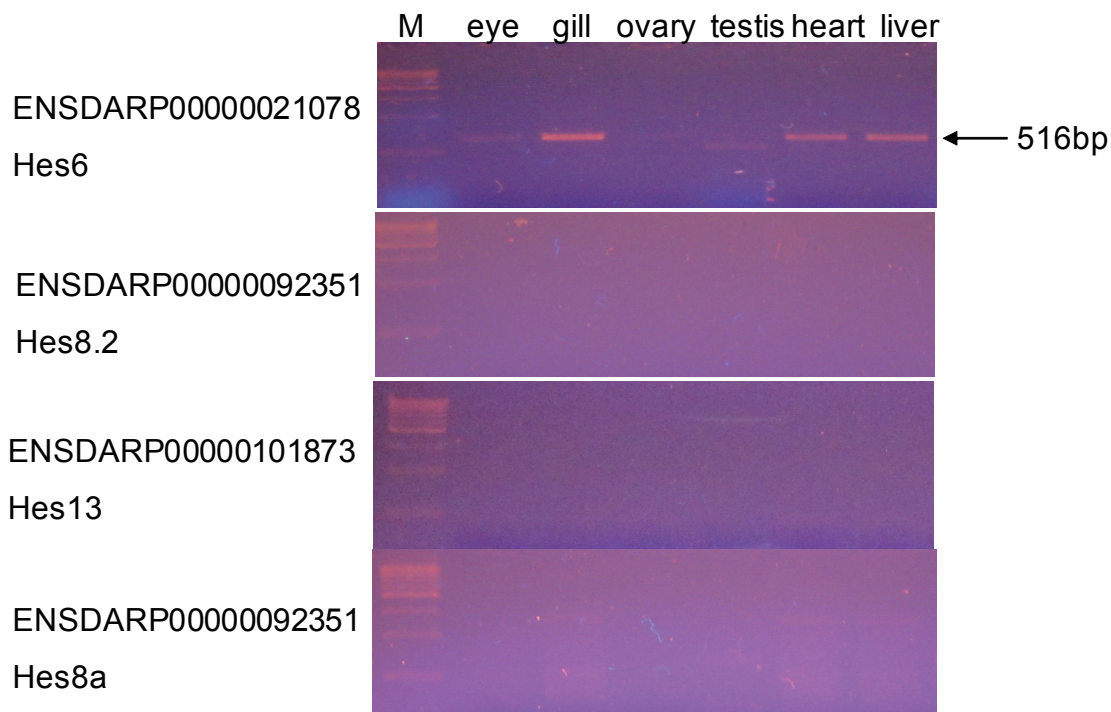

## HES6 expression in zebrafish by RT-PCR

(b)

Supplement: Figure S6 — HES2 (a) and HES6 (b) expression in zebrafish tissues detected by RT-PCR. (PDF) [file pone.0040649.s006.pdf]
