# Supplementary material for: Comparative and Evolutionary Analysis of the HES/HEY Gene Family Reveal Exon/Intron Loss and Teleost Specific Duplication Events
Source: PLoS One. 2012 Jul 13;7(7):e40649. doi: 10.1371/journal.pone.0040649 (PMC3396596; doi:10.1371/journal.pone.0040649)

A

HES/HEY-ML(JTT+I+G)

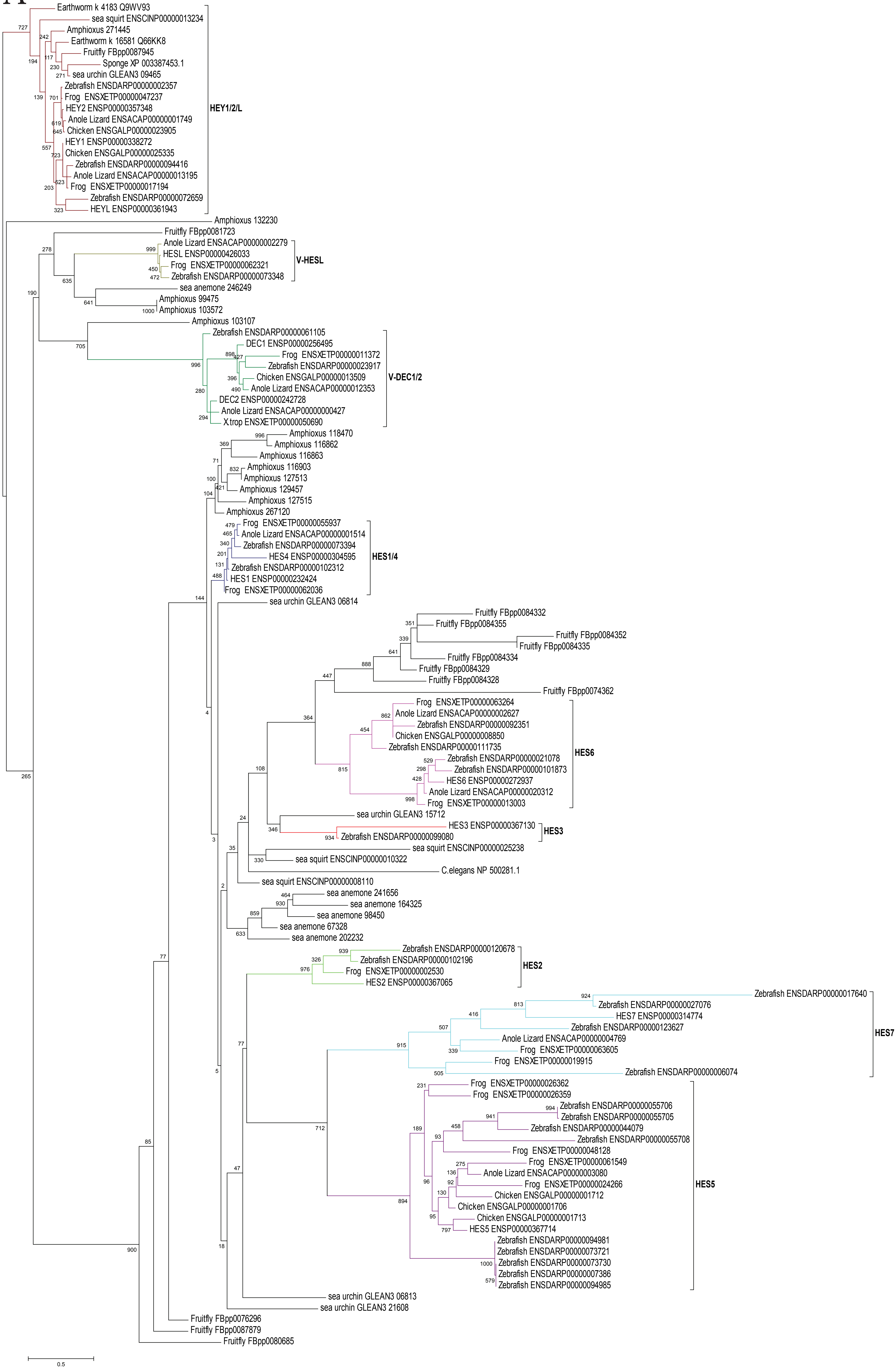

B

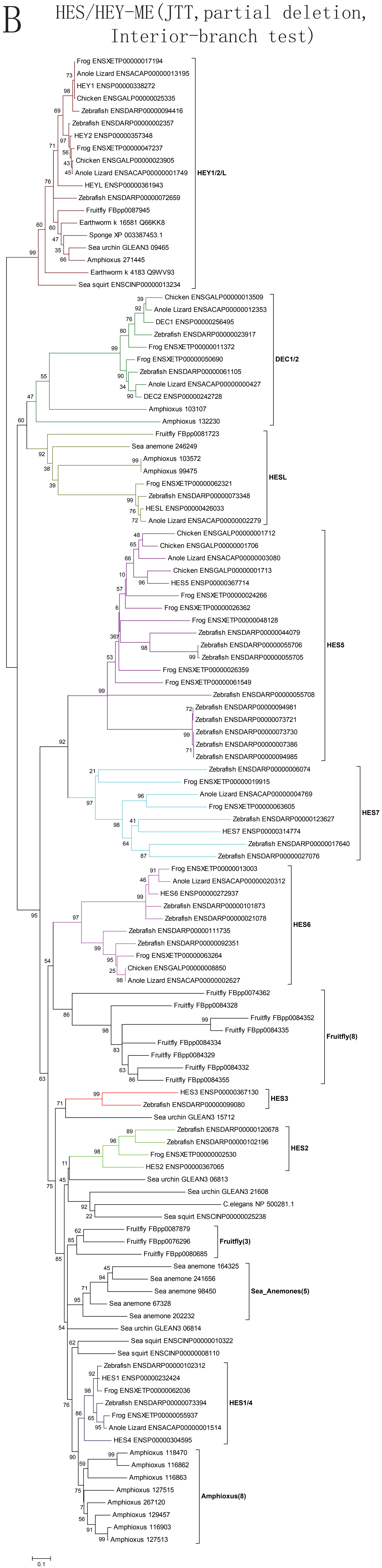
HES/HEY-ME(JTT,partial deletion, Interior-branch test)
Frog ENSXETP00000017194
Anole Lizard ENSACAP00000013195
HEY1 ENSP00000338272
Chicken ENSGALP00000025335
Zebrafish ENSDARP00000094416
Zebrafish ENSDARP00000002357
HEY2 ENSP00000357348
Frog ENSXETP00000047237
Chicken ENSGALP00000023905
Anole Lizard ENSACAP00000001749
HEYL ENSP00000361943
Zebrafish ENSDARP00000072659
Fruitfly FBpp0087945
Earthworm k 16581 Q66KK8
Sponge XP 003387453.1
Sea urchin GLEAN3 09465
Amphioxus 271445
Earthworm k 4183 Q9WV93
Sea squirt ENSCINP00000013234
HEY1/2/L
Chicken ENSGALP00000013509
Anole Lizard ENSACAP00000012353
DEC1 ENSP00000256495
Zebrafish ENSDARP00000023917
Frog ENSXETP00000011372
Frog ENSXETP00000050690
Zebrafish ENSDARP00000061105
Anole Lizard ENSACAP00000000427
DEC2 ENSP00000242728
Amphioxus 103107
Amphioxus 132230
DEC1/2
Fruitfly FBpp0081723
Sea anemone 246249
Amphioxus 103572
Amphioxus 99475
Frog ENSXETP00000062321
Zebrafish ENSDARP00000073348
HESL ENSP00000426033
Anole Lizard ENSACAP00000002279
HESL
Chicken ENSGALP00000001712
Chicken ENSGALP00000001706
Anole Lizard ENSACAP00000003080
Chicken ENSGALP00000001713
HES5 ENSP00000367714
Frog ENSXETP00000024266
Frog ENSXETP00000026362
Frog ENSXETP00000048128
Zebrafish ENSDARP00000044079
Zebrafish ENSDARP00000055706
Zebrafish ENSDARP00000055705
Frog ENSXETP00000026359
Frog ENSXETP00000061549
Zebrafish ENSDARP00000055708
Zebrafish ENSDARP00000094981
Zebrafish ENSDARP00000073721
Zebrafish ENSDARP00000073730
Zebrafish ENSDARP00000007386
Zebrafish ENSDARP00000094985
HES5
Zebrafish ENSDARP00000006074
Frog ENSXETP00000019915
Anole Lizard ENSACAP00000004769
Frog ENSXETP00000063605
Zebrafish ENSDARP00000123627
HES7 ENSP00000314774
Zebrafish ENSDARP00000017640
Zebrafish ENSDARP00000027076
HES7
Frog ENSXETP00000013003
Anole Lizard ENSACAP00000020312
HES6 ENSP00000272937
Zebrafish ENSDARP00000101873
Zebrafish ENSDARP00000021078
Zebrafish ENSDARP00000111735
Zebrafish ENSDARP00000092351
Frog ENSXETP00000063264
Chicken ENSGALP00000008850
Anole Lizard ENSACAP00000002627
HES6
Fruitfly FBpp0074362
Fruitfly FBpp0084328
Fruitfly FBpp0084352
Fruitfly FBpp0084335
Fruitfly FBpp0084334
Fruitfly FBpp0084329
Fruitfly FBpp0084332
Fruitfly FBpp0084355
Fruitfly(8)
HES3 ENSP00000367130
Zebrafish ENSDARP00000099080
HES3
Sea urchin GLEAN3 15712
Zebrafish ENSDARP00000120678
Zebrafish ENSDARP00000102196
Frog ENSXETP00000002530
HES2 ENSP00000367065
HES2
Sea urchin GLEAN3 06813
Sea urchin GLEAN3 21608
C.elegans NP 500281.1
Sea squirt ENSCINP00000025238
Fruitfly FBpp0087879
Fruitfly FBpp0076296
Fruitfly FBpp0080685
Fruitfly(3)
Sea anemone 164325
Sea anemone 241656
Sea anemone 98450
Sea anemone 67328
Sea anemone 202232
Sea_Anemones(5)
Sea urchin GLEAN3 06814
Sea squirt ENSCINP00000010322
Sea squirt ENSCINP00000008110
Zebrafish ENSDARP00000102312
HES1 ENSP00000232424
Frog ENSXETP00000062036
Zebrafish ENSDARP00000073394
Frog ENSXETP00000055937
Anole Lizard ENSACAP00000001514
HES4 ENSP00000304595
HES1/4
Amphioxus 118470
Amphioxus 116862
Amphioxus 116863
Amphioxus 127515
Amphioxus 267120
Amphioxus 129457
Amphioxus 116903
Amphioxus 127513
Amphioxus(8)
0.1

C HES/HEY-NJ(JTT, partial deletion, Interior-branch test)

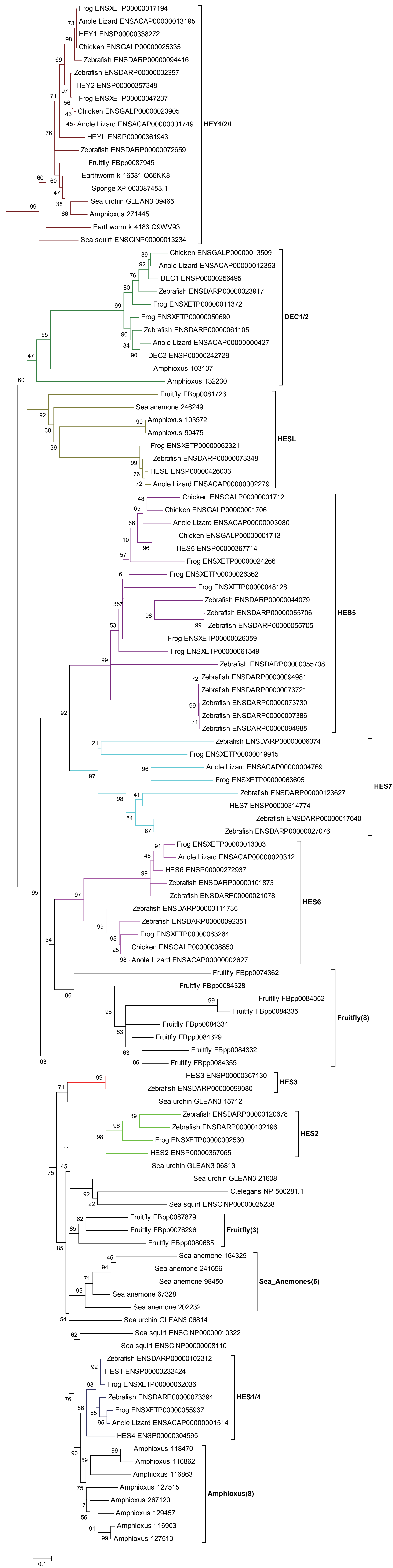

Supplement: Figure S3 — Phylogenetic trees by some other methods. (A) Phylogenetic ML tree of HES/HEY genes (JTT+I+G). (B)Phylogenetic ME (JTT) tree of HES/HEY genes. (C) Phylogenetic NJ (JTT) tree of HES/HEY genes. (PDF) [file pone.0040649.s003.pdf]
