# Supplementary material for: Comparative and Evolutionary Analysis of the HES/HEY Gene Family Reveal Exon/Intron Loss and Teleost Specific Duplication Events
Source: PLoS One. 2012 Jul 13;7(7):e40649. doi: 10.1371/journal.pone.0040649 (PMC3396596; doi:10.1371/journal.pone.0040649)
Supplement: Figure S4 — Exon-intron structure of human HES/HEY genes. The color representations have described in Figure 3. The four groups of HEY1/2/L, DEC1/2, HESL, and HES1-7 were circled by purple boxes. (PDF) [file pone.0040649.s004.pdf]

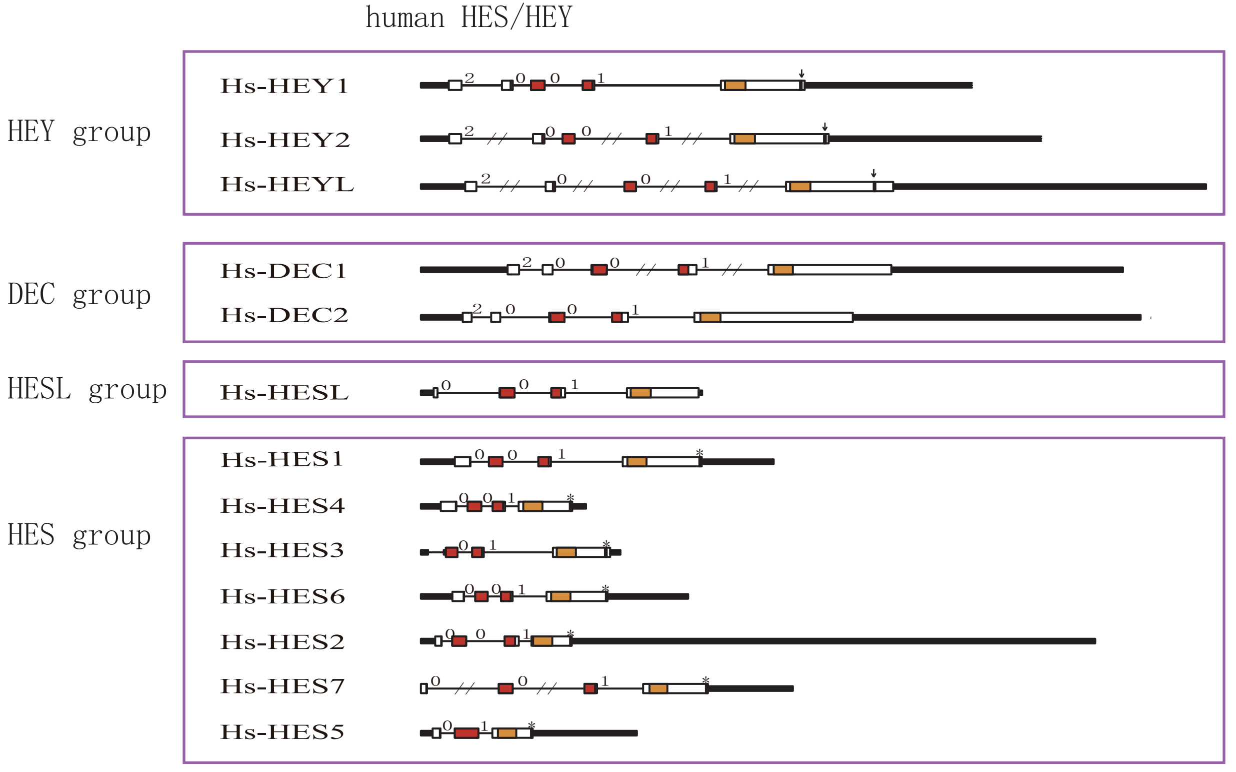
human HES/HEY
HEY group
Hs-HEY1
Hs-HEY2
Hs-HEYL
DEC group
Hs-DEC1
Hs-DEC2
HESL group
Hs-HESL
HES group
Hs-HES1
Hs-HES4
Hs-HES3
Hs-HES6
Hs-HES2
Hs-HES7
Hs-HES5
