# Supplementary material for: Comparative and Evolutionary Analysis of the HES/HEY Gene Family Reveal Exon/Intron Loss and Teleost Specific Duplication Events
Source: PLoS One. 2012 Jul 13;7(7):e40649. doi: 10.1371/journal.pone.0040649 (PMC3396596; doi:10.1371/journal.pone.0040649)
Supplement: Table S2 — 13 HES/HEY genes in Fruit fly. (DOC) [file pone.0040649.s008.doc]

**Table.S2 Fruitfly 13 HES/HEY genes**

| Human homolog | Gene symbol | NCBI gene ID | Full name | Ensembl gene ID | Ensembl protein ID | Protein length | Location(chr:start-end:strand) |
| --- | --- | --- | --- | --- | --- | --- | --- |
| HEY1/HEY2/HEYL | Hey | 35764 | Hey Hairy/E(spl)-related with YRPW motif | FBgn0027788 | FBpp0087945 | 425 | Chr2R: 3,882,941-3,886,078:-1 |
| DEC1/2 | cwo | 44669 | cwo clockwork orange | FBgn0259938 | FBpp0081723 | 698 | Chr3R: 6,213,985-6,226,072:1 |
| HES1/4 | h | 38995 | h hairy | FBgn0001168 | FBpp0076296 | 337 | Chr3L: 8,668,859-8,672,339:1 |
| HES1/4 | Side | 35168 | Side similar to Deadpan | FBgn0032741 | FBpp0080685 | 507 | Chr2L: 19,027,712-19,030,787:1 |
| HES1/4 | dpn | 35800 | dpn deadpan | FBgn0010109 | FBpp0087879 | 435 | Chr2R: 4,116,476-4,119,832:-1 |
| HES6 | Her | 32800 | Her HES-related | FBgn0030899 | FBpp0074362 | 149 | ChrX: 18,098,576-18,099,153:-1 |
| HES6 | HLHmdelta | 43150 | HLHmdelta E(spl) region transcript mdelta | FBgn0002734 | FBpp0084328 | 173 | Chr3R: 21,823,343-21,824,358:1 |
| HES6 | HLHmgamma | 43151 | HLHmgamma E(spl) region transcript mgamma | FBgn0002735 | FBpp0084329 | 205 | Chr3R: 21,825,580-21,826,421:1 |
| HES6 | HLHm3 | 43156 | HLHm3 E(spl) region transcript m3 | FBgn0002609 | FBpp0084332 | 224 | Chr3R: 21,847,437-21,848,858:1 |
| HES6 | HLHm7 | 43160 | HLHm7 E(spl) region transcript m7 | FBgn0002633 | FBpp0084334 | 186 | Chr3R: 21,862,760-21,863,482:1 |
| HES6 | E(spl) | 43161 | E(spl) Enhancer of split | FBgn0000591 | FBpp0084335 | 179 | Chr3R: 21,866,046-21,866,585:1 |
| HES6 | HLHm5 | 43158 | HLHm5 E(spl) region transcript m5 | FBgn0002631 | FBpp0084352 | 178 | Chr3R: 21,854,572-21,855,457:-1 |
| HES6 | HLHmbeta | 43152 | HLHmbeta E(spl) region transcript mbeta | FBgn0002733 | FBpp0084355 | 195 | Chr3R: 21,830,648-21,831,745:-1 |

* Location is on the BDGP5.25.62
