# Supplementary material for: Comparative and Evolutionary Analysis of the HES/HEY Gene Family Reveal Exon/Intron Loss and Teleost Specific Duplication Events
Source: PLoS One. 2012 Jul 13;7(7):e40649. doi: 10.1371/journal.pone.0040649 (PMC3396596; doi:10.1371/journal.pone.0040649)
Supplement: Table S3 — 28 HES/HEY genes in zebrafish (Zv9). (DOC) [file pone.0040649.s009.doc]

**Table S3 Zebrafish 28 HES/HEY genes** (Zv9)

| Human homolog | Gene symbol | NCBI gene ID | Full name | Ensembl gene ID | Ensembl protein ID | Protein length | Location(chr:start-end:strand) |
| --- | --- | --- | --- | --- | --- | --- | --- |
| HEY1 | hey1 | 58008 | hey1 hairy/enhancer-of-split related with YRPW motif 1 | ENSDARG00000070538 | ENSDARP00000094416 | 317 | Chr19:33042965:33047694:-1 |
| HEY2 | hey2 | 58146 | hey2 hairy/enhancer-of-split related with YRPW motif 2 | ENSDARG00000013441 | ENSDARP00000002357 | 324 | Chr20:39614524:39622047:-1 |
| HEYL | heyl | 335134 | heyl hairy/enhancer-of-split related with YRPW motif-like | ENSDARG00000055798 | ENSDARP00000072659 | 310 | Chr19:33929338:33936055:1 |
| DEC1 | bhlhe40 | 324413 | bhlhe40 basic helix-loop-helix family, member e40 | ENSDARG00000004060 | ENSDARP00000023917 | 403 | Chr11:36600441:36604112:-1 |
| DEC2 | bhlhe41 | 563771 | bhlhe41 basic helix-loop-helix family, member e41 | ENSDARG00000041691 | ENSDARP00000061105 | 421 | Chr18:15785020:15789333:1 |
| HESL | helt | 404275 | helt hey-like transcription factor | ENSDARG00000056400 | ENSDARP00000073348 | 270 | Chr1:16335287:16336940:1 |
| HES1 | her9 | 140613 | her9 hairy-related 9 | ENSDARG00000056438 | ENSDARP00000073394 | 291 | Chr23:23693662:23695447:-1 |
| HES1 | her6 | 30288 | her6 hairy-related 6 | ENSDARG00000006514 | ENSDARP00000102312 | 331 | Chr6:36329967:36332018:-1 |
| HES6 | hes6 | 373116 | hes6 hairy and enhancer of split 6 | ENSDARG00000019335 | ENSDARP00000021078 | 226 | Chr2:48417314:48418868:1 |
| HES6 | her8.2 | 565269 | her8.2 hairy-related 8.2 | ENSDARG00000069675 | ENSDARP00000092351 | 223 | Chr15:7103816:7106497:1 |
| HES6 | her13 | 550600 | her13 hairy-related 13 | ENSDARG00000007097 | ENSDARP00000101873 | 224 | Chr15:7114862:7117817:1 |
| HES6 | her8a | 323656 | her8a hairy-related 8a | ENSDARG00000016363 | ENSDARP00000111735 | 253 | Chr7:27555809:27562348:-1 |
| HES2 | LOC559147 | 559147 | LOC559147 novel protein similar to vertebrate hairy and enhancer of split 2 | ENSDARG00000074897 | ENSDARP00000102196 | 195 | Chr8:49077686:49082343:-1 |
| HES2 | hes2.2 | 751634 | hes2.2 hairy and enhancer of split 2.2 | ENSDARG00000068168 | ENSDARP00000120678 | 191 | Chr8:49052584:49053873:-1 |
| HES3 | her3 | 30289 | her3 hairy-related 3 | ENSDARG00000076857 | ENSDARP00000099080 | 253 | Chr8:48790624:48791866:-1 |
| HES7 | her7 | 58132 | her7 hairy and enhancer of split related-7 | ENSDARG00000017917 | ENSDARP00000006074 | 206 | Chr5:71711885:71713200:1 |
| HES7 | her11 | 445409 | her11 hairy-related 11 | ENSDARG00000002707 | ENSDARP00000017640 | 274 | Chr14:31408114:31410596:-1 |
| HES7 | her1 | 30287 | her1 hairy-related 1 | ENSDARG00000014722 | ENSDARP00000027076 | 328 | Chr5:71693373:71699778:-1 |
| HES7 | her5 | 30285 | her5 hairy-related 5 | ENSDARG00000008796 | ENSDARP00000123627 | 227 | Chr14:31412619:31414295:1 |
| HES5 | her12 | 402914 | her12 hairy-related 12 | ENSDARG00000032963 | ENSDARP00000044079 | 155 | Chr23:21739064:21741127:-1 |
| HES5 | her15.2 | 678530 | her15.2 hairy and enhancer of split-related 15.2 | ENSDARG00000054560 | ENSDARP00000055705 | 149 | Chr11:43102143:43103588:-1 |
| HES5 | her15.1 | 359836 | her15.1 hairy and enhancer of split-related 15.1 | ENSDARG00000054562 | ENSDARP00000055706 | 149 | Chr11:43089905:43091345:1 |
| HES5 | her2 | 30300 | her2 hairy-related 2 | ENSDARG00000038205 | ENSDARP00000055708 | 108 | Chr11:43073539:43077202:-1 |
| HES5 | LOC100148329 | 100148329 | LOC100148329 hairy-related 4.2-like | ENSDARG00000009822 | ENSDARP00000073721 | 152 | Chr23:21756163:21757930:-1 |
| HES5 | LOC100149863 | 100149863 | LOC100149863 hairy-related 4.1-like | ENSDARG00000056732 | ENSDARP00000073730 | 152 | Chr23:21746699:21747756:-1 |
| HES5 | her4.2 | 30301 | her4.2 hairy-related 4.2 | ENSDARG00000056729 | ENSDARP00000094981 | 152 | Chr23:21763345:21765164:-1 |
| HES5 | her4.3 | 792198 | her4.3 hairy-related 4.3 | ENSDARG00000070770 | ENSDARP00000094984 | 152 | Chr23:21753405:21755282:1 |
| HES5 | LOC100148329 | 100148329 | LOC100148329 hairy-related 4.2-like | ENSDARG00000094426 | ENSDARP00000122768 | 152 | Chr23:21749296:21751084:1 |

* Location is on the Zv9
