# Supplementary material for: Comparative and Evolutionary Analysis of the HES/HEY Gene Family Reveal Exon/Intron Loss and Teleost Specific Duplication Events
Source: PLoS One. 2012 Jul 13;7(7):e40649. doi: 10.1371/journal.pone.0040649 (PMC3396596; doi:10.1371/journal.pone.0040649)
Supplement: Table S7 — The excluded sequences in the phylogenetic analysis. (DOC) [file pone.0040649.s013.doc]

> Sea_Anemones _28948

ARKRRRGLIEKKRRDRINRCLVELRRLVPTALEKEGSSKLEKAEILHLTVEHLKWLRSTS

GQSRSDVTDYRAAGFQECLTEVAKYMATINND

> Amphioxus _65983

MSSGRRQRQACGKLEKAEILEMTVEYIRYLQEERRHARHLFGAQYWTTSYAFYRMGYGDC

MRDVHNYFGSIGTGGVSTPDVSGHNHLMNYLQHKATLSGASPIGISQSDRRGYVHGDARL

RQSYANSSCLSRPEGLRSQGTSPTSCSHIHLEHAQNITDVPKTTQYLQTPRSSNSPDLDG

LETSYYSEFESQREHTMK

> Amphioxus _129955

MSEGNQQSQANTGGTTPQQQPQTDWRTIADAAANIPNTMYVSRAVRVIRHSEDMAKVFKQ

IDKLINRTERKFPALADLEWSRGPQGKMGEMGPNGPVSVGPPGPPGEKGAMGPAGPVSVG

PPGPPGEKGPAGPVSVGPPGPPGEKGAMGPAGPASVGPPGPPEEKGAMGPAGPVSVGPPG

PPGEKGAMGPAGPVSVGPPGPPGDKGAMGPAGPVSVGPPGPPGEKGAMGPPGPPGEKGAM

GPAGPPGEKGAMGPTGPSGEKGAVGPAGPLGKTGPIGPAGPVSFGRPGPPGPPGLPGNSF

CPGLAERASCPEGYTMWRETCYKAFNEYKFVGTFSEATETCRKDGGTLAMPRDAETNRFL

VTLERNKGSFYWIGLHDQRMEGTFEWVDGSALGRYNSWTVEQPDNKDGDEDCVAFSSHSG

RGHWYDEKCRHIEYIFCQVAPGRIGTGIAFSPTRDPNKTTAGIKTTEPVSRNQAWINSRC

YTQSFDAYFAGSAKLEKAEILQMTVDYLKMLAAKGYHAYDDHFIDYRGIGFRECANEVAR

YMVTIEGLDIQDPLRIRLMNHLQCIAAQREAQVVAAAGMRHTSPWNVNGVTAPHPQFASV

QAAPDAQGLLHATSLHEPPRQPAAIACSTPNTYLKTSAHLPVAASVPASSTAQISPTLIS

STQFPVTFNTLHLSPTGYGTTSTTQSQIITKPYRPWGSEVNAY

>Chicken_ENSGALP00000034328

MKRLCEESSSDTESDGTIDVGKEEEYSHIPRSVSPTTTSQIQARKKRRGGKELHFLLDKN

LRQCPETGDGALGVFRRVWEEGARQRQGSSKLEKAEILQMTVDHLKMLHATGGAGFLDAR

ALAVDYRSIGFRECLTEVVRYLGVLEGQSTADPIRLRLLSHLNNYVAEMEPSPAAASLLP

VQTWPWSFLHSPVPVPRREAAPAPLLVTASYPRLAARPAPVRRVPADMLPSHRGPLPGRM

ASSARRARGAPSASTAAAAPRMPSPSGAPRGGSAISALLFSPAGVPLPAAYAAPTVLGAA

AQGPALRMGAARLCRSWATEIGAF

>Chicken_ENSGALP00000039369

KQKRKEEKEKRNKKKEEKRKTDEKKKKEGEKRRREEEKSKKNKSSRHSKLEKADILEMTV

KHLRNLQRAQMAAALSADPSVLGKYRAGFNECMNEVTRFLSTCEGVNADVRARLLGHLSA

CLGQIVAMNYLPPPPAGQPAHLAQPLHVQLPPTTTGAVPVPCKLEPTEALSPKVYGGFQL

VPATDGQFAFLIPNPAFPPGSGPVIPLYANANVPVSTSGGSGNASTTPSASPVQGLTSFG

HSVVPASQAGSPIAERRESVWRPW

> Anole_Lizard _ENSACAP00000003987

SSRHSKLEKADILEMTVKHLRGLQRAQRSAVLNTDPSVLGKYRAGFSECVNEVTRFLSTC

EGVNAEVRTRLLGHLAGCMSQISAIRFPAAPQQPQPPPAAPGSQPVASFGQALVQVPATG

ALPKAGSGAPCKTVPREAPQVFGGFHLVPASDGQFAFLIPNAAFAPHGSNAAAPLFASTG

AGSGGAPPSTNAVSPGSGAPSLPSDSVWRPW

>Earthworm|a_19463|P26439|3 beta-hydroxysteroid dehydrogenase/Delta 5-->4-isomerase type 2 [Homo sapiens]

LLTVSTGNGSRLHTVCLRPNVMYGEGDPYYVINGLKSAKQNDGILVRIGDGSAKFQQAYVGNVAWAHLCAVRAVQLDPGLGGRNFFVTDDTPLMNTFTFMKPFLRSRGFDLSDNSIPYGLVYAVYFWIDWILWALKPIWKVNLEVALPSLVYVNHTLYFNRRNAEEDLRYKPLYGYQTSLAKSLEFYKRVPLNK

>Earthworm|a_16410|Q8AVU4|Transcription factor HES-1-B [Xenopus laevis]

SNESRYSKMEKVDILEMTVRYLQDLRRSFPACAGSPPSQSLTAKYIEGYSECASQVGQYLSLTKELSQDIRQRLSQHLVDSLRRTTAPTRLASPLGEAQAAGILSIGQAIDSRSSFQSVVNTDCASTMLRTSVQSPAILSTKFAVLETETGSLS

>Earthworm|a_7910|Q7KM13|Hairy/enhancer-of-split related with YRPW motif protein [Drosophila melanogaster]

TCYCFSVQLCLDFNSNDGICQTCFFQESSYSKMEKADILEMTVAHLKSLQHRHPENDPKVRDHDPATRYVLGFRECATEVEKYLTTLNTVDRPGVEVLSTLIQYLRDRTEDVQKAVRSGSDQLHPVMESSGFGRAESLMQQLHAAHQPSKIVEYECNEFQPSTGANCFRMSDSSAPDSATNAATRYILGQLPYLQHPYAGLTSSLTISEQSHFATSATSLTLARREQAQSPTHGDEFPRALGVRIPRASGMFQHLRDSQISPDSGISLDSPIQVYRSKSVKSESVVEFSSMSGCKVKPRSQLETMESGPSSDHPWRPW
