# Supplementary material for: Comparative and Evolutionary Analysis of the HES/HEY Gene Family Reveal Exon/Intron Loss and Teleost Specific Duplication Events
Source: PLoS One. 2012 Jul 13;7(7):e40649. doi: 10.1371/journal.pone.0040649 (PMC3396596; doi:10.1371/journal.pone.0040649)
Supplement: Table S8 — The primers used for the RT-PCR assays. (DOC) [file pone.0040649.s014.doc]

**Table S8 The primers used for the RT-PCR assays**

|  | forward | reverse |
| --- | --- | --- |
| ENSDARP00000120678 | 5-atgactcccagcgccaccgctg-3 | 5-ctaccaaggtctccagactt-3 |
| ENSDARP00000102196 | 5-atggcgtcttcttcatcgtc -3 | 5-tcaccacggctccacatgtcctcagctg-3 |
| ENSDARP00000006074 | 5-atgaaaatcctggcacagac-3 | 5- ttaaggccaaggtctccaga-3 |
| ENSDARP00000027076 | 5-atgccatgtgcgtccatccc-3 | 5-tcaccagggtctccacaaag-3 |
| ENSDARP00000017640 | 5- atgaagagcacaccgacttt-3 | 5-tcagctccatggtctccagg-3 |
| ENSDARP00000023627 | 5-atgctcaaaatctcagactg-3 | 5-ctaccagggcctccagac-3 |
| ENSDARP00000021078 | 5-atggagaacgccgaggtgttgg-3 | 5- ctaccagggtctccaaatggactt-3 |
| ENSDARP00000092351 | 5-atgaagagcacaccgacttt-3 | 5-tcaccagggcctccacatctgtaga-3 |
| ENSDARP00000101873 | 5-atggtccacgctgctgaactt-3 | 5- ctaccatggcctccacatgaa-3 |
| ENSDARP00000092351 | 5-atgacggcctccaacatggg-3 | 5-tcaccagggcctccacatggaaggat-3 |
| β-actin | 5- gatctggcatcacaccttc-3 | 5-ctcatagatgggcacggtg-3 |
